# Webcams can be used to study eye movements during reading

**DOI:** 10.3758/s13428-026-03199-3

**Published:** 2026-10-21

**Authors:** Marina Serrano-Carot, Bernhard Angele, Hemu Xu, Martin R. Vasilev

**Affiliations:** 1 Centro de Investigación Nebrija en Cognición (CINC), Universidad Nebrija, Madrid, Spain; 2 Department of Experimental Psychology, University College London, 26 Bedford Way, London, WC1H 0AP UK

**Keywords:** Eye movements, Reading, Eye tracking, Webcams, Online testing

## Abstract

Eye movements have revealed a wealth of information about how readers process written language. Modern eye trackers can record eye movements in the lab with high accuracy. However, such equipment is expensive, and participant testing is labour-intensive. Kaduk et al. (Behav Res Methods 56(5):5002–5022, 2023) recently reported that webcam eye tracking may be a viable alternative, showing that webcams achieved correlations with laboratory eye trackers of ~0.83 during a free-viewing task. We conducted three experiments to test whether webcams can be used to study eye movements during reading. Participants read short sentences with a target word frequency manipulation while their eye movements were recorded with both an EyeLink system and a webcam in the laboratory (co-registration, Experiments 1 and 2) or online with their own webcam (online study, Experiment 3). In the lab, the webcam-based system recorded raw gaze positions that were highly correlated with the EyeLink (median *r* = 0.805), replicating Kaduk et al.’s results. We also found evidence for word frequency effects in the webcam data, but there was more noise than in the EyeLink data. In the online study, data quality was much poorer, but we still found reliable effects that were consistent with those observed in the laboratory. In summary, webcam-based eye tracking may be viable for studying reading, especially under laboratory conditions. However, obtaining useful data may require collecting many more observations when using webcams than with high-accuracy eye trackers.

**Supplementary Information:**

The online version contains supplementary material available at https://doi.org/10.3758/s13428-026-03199-3.

Learning to read and write was a fundamental achievement in human history which paved the way for the development of a knowledge-based modern society. The ability to decode information that others have written, along with the ability to encode new written information, underlies almost every area of human activity today. Much of what we know about language processing during reading comes from the study of eye movements. The importance of eye movements for vision was clear even to ancient scholars (Wade, 2010), but, until the invention of eye trackers, there was no method to study eye movements beyond introspection and direct observation of a participant while they were reading. Introspection is unreliable for many reasons, but, in the case of eye movements, it is particularly unsuited because saccadic suppression makes it impossible to directly perceive one’s own eye movements (Matin, 1974). Direct observation of eye movements in other people does not have this issue, but it is very difficult to do it accurately without technical instruments.

Huey (1908) described some of the early attempts made by researchers such as Javal, Dodge, and Erdmann to record eye movements using devices attached to the eyeball and photographic methods (see also Wade & Tatler, 2008). Researchers like Yarbus (1967; see also Tatler et al., 2010; Richardson & Spivey, 2008) explored these new eye-tracking technologies in order to characterise eye movements in a variety of tasks, such as scene viewing. However, it was not until the introduction of infrared-based eye trackers in the 1970s that eye movements emerged as a method for observing the moment-to-moment evolution of language processing during reading. The use of infrared light allowed for much better illumination of the eyes than with visible light, and highly sensitive photodiodes enabled researchers to track the relative positions of the light reflections on the outer and inner parts of the eye with high precision. Such dual Purkinje image (DPI) eye trackers (Cornsweet & Crane, 1973) allowed researchers to record both the temporal and spatial characteristics of eye movements with high precision and made it possible to calculate word-based measures, such as average fixation duration on a target word (e.g., Ehrlich & Rayner, 1981). Moreover, the use of DPI eye trackers allowed researchers to change the stimuli presented on the computer display depending on the participant’s gaze position (gaze-contingent display changes). This led to the creation of novel experimental techniques, such as the moving window paradigm, where all text outside of a certain area around the fixation position is masked (McConkie & Rayner, 1975), and the boundary paradigm, where the parafoveal preview that readers receive of one specific target word is manipulated before readers fixate the target word directly. Once a reader’s gaze crosses the invisible boundary between the pre-target word and the target word, the display is changed to show the actual target word, making it possible to manipulate the parafoveal information that a reader receives about a word without the participant being consciously aware of the manipulation (Rayner, 1975a, 1975b). Modern video-based infrared eye trackers, such as SR Research’s EyeLink 1000 Plus, have replaced the photodiodes with high-speed video cameras which have much higher tolerance for participant head movements. Using these and other eye-tracking techniques, we have learned much about how readers process words and sentences (for a review, see Rayner, 1998, 2009; as well as Schotter et al., 2012; Clifton et al., 2016).

Eye-tracking research using video-based infrared eye trackers has many advantages: It is non-invasive, the methodology is relatively easy to learn and works on most participants, and it can be set up in any laboratory space that is away from direct sunlight, has enough space for participants to be seated at the recommended monitor and camera distances, and has an electrical outlet. Infrared-based eye tracking can also be combined with other methods such as electroencephalography (EEG, Dimigen et al., 2011; see also Degno et al., 2021), magnetoencephalography (MEG, Pan et al., 2021), and functional magnetic resonance imaging (fMRI, Choi et al., 2014). However, as Angele and Duñabeitia (2024) showed, the vast majority of eye-tracking research in reading is conducted in only a handful of countries, mainly in North America, Western Europe, and, most recently, China. They argue that this is likely due to the cost of a modern, high-precision eye tracker, which, although moderately priced relative to other methods such as fMRI, MEG, and EEG, still represents a substantial investment for many researchers in countries where research funding is difficult to obtain. For this reason, many languages and writing systems are rarely studied or not studied at all using eye movements.

One possible solution for this issue comes from online research. During the COVID-19 pandemic, very little eye-tracking research could be done in the laboratory, but many researchers started doing behavioural language research online, such as studies using the lexical decision task. Paid platforms such as PsychoPy/Pavlovia (Bridges et al., 2020; Peirce et al., 2022) and Gorilla (Anwyl-Irvine, Dalmaijer, et al., 2020a, 2020b; Anwyl-Irvine, Massonnié, et al., 2020a, 2020b) and open-source solutions such as JATOS (Lange et al., 2015) or lab.js (Henninger et al., 2022) take advantage of the performance of JavaScript in the browser in order to provide reasonably high-precision and accuracy stimulus presentation and response collection on virtually any PC or, in some cases, mobile device. For example, Angele et al. (2022) found that masked word priming effects can be observed in online experiments for stimulus presentation times as low as 33 ms and 50 ms. This suggests that even experiments that depend on very accurate stimulus timing can be performed online and yield results equivalent to a laboratory study. This not only enabled researchers to continue collecting data during the pandemic but also opened the possibility of reaching participants who would not easily be able to go to the laboratory.

While there are some paradigms for the study of reading without recording eye movements, such as self-paced reading or the maze task (Forster et al., 2009), the reaction times from those tasks cannot provide an “online” account of ongoing processing in the same way that eye movements can. Is it possible then to track eye movements in an online experiment? In principle, most mobile devices and laptop computers today are equipped with front-facing webcams, which could be used to monitor a participant’s eye position. In practice, calculating the gaze position on the screen from a webcam image faces a number of challenges: First, webcams are optimised for recording an acceptable image of the user for videocalls, using visible light. There is no dedicated infrared illuminator, so any recording needs to rely on the illumination produced by the screen and the ambient lighting. In an online experiment, we can control what is on the screen, but we have no control over the lighting environment. We can ask the participant to move to a well-lit area and avoid backlighting, but our ability to control whether the participant is following these instructions is limited. This alone makes identifying features such as the eyes, the pupils, or the corneal reflection much more difficult. In fact, since there is no dedicated illuminator, there may not be a consistent corneal reflection to track, so a webcam-based eye-tracking method likely must rely on the orientation of the pupil in relation to the rest of the face to identify gaze position. Additionally, both the spatial (usually 720p or 1,280 × 720) and the temporal resolution (30 frames per second, fps) of typical webcams is quite low. With built-in webcams, there is the additional issue that these devices are usually optimised to be very small and thin, requiring the use of small, single-lens set-ups and small sensors that limit image quality. In terms of the spatial resolution, it is therefore clear that the webcam eye-tracking data will be much less precise and accurate than the data recorded by a laboratory-based eye tracker. To compensate for this, an online eye-tracking application will have to apply some data processing, often using machine learning algorithms, to arrive at acceptable data quality. To maintain participant privacy and avoid having to upload and store videos of the participant’s face, this post-processing must be done locally (and in the browser), limiting the computational resources available.

In terms of the temporal resolution, or sampling rate, there is the concern that it may not be possible to accurately detect the main components of eye movements during reading: saccades, which are fast, ballistic eye movements that move the gaze from one part of the visual scene to another, and fixations, which are the periods of relative gaze position stability in between saccades during which visual information about the text is obtained. Based on the Nyquist–Shannon sampling theorem (Shannon, 1949), a sampling rate of 148 Hz would be required to capture all the information present in the gaze velocity signal, which is then used to detect saccades and fixations (Andersson et al., 2010; Angele et al., 2025). A sampling rate of 30 fps (or Hz) is significantly lower than this. However, even if some velocity information is lost due to a low sampling rate, the resulting detected fixation durations might still contain useful information about the reading process. Angele et al. (2025) simulated low sampling rates by eliminating samples from data collected with an EyeLink Portable Duo at sampling rates between 250 and 2,000 Hz. Even at simulated sampling rates as low as 31.25 Hz, they were able to replicate the word frequency effect on fixation times in reading, i.e., the observation that a word that occurs in the language frequently is, on average, fixated for less time than a word that occurs very rarely (Rayner & Duffy, 1986). The word frequency effect is considered a “benchmark” effect in the study of reading, as it has been reliably found in a large number of studies (Rayner, 1998) and is considered evidence of the impact of cognitive processes (learning) on eye movements (Brysbaert et al., 2018). Generally, the estimates of the size of the frequency effect in Angele et al.’s (2025) study were consistent across sampling rates (5–10 ms for first fixation duration and 35–45 ms for gaze duration). This demonstrates that even eye movements recorded at only around 30 Hz can contain useful information about ongoing cognitive processing. In principle, this should also be true for eye movements recorded with a webcam.

Recent work has further tested this assumption in applied contexts. For example, Van Boxtel et al. (2024) compared WebGazer-based online eye tracking (≈14 Hz sampling, Papoutsaki et al., 2016; see below for a more extensive discussion) with laboratory recordings (≈430 Hz) in individuals with aphasia and control participants. They found that webcam eye tracking reproduced the same overall group-level differences in sentence comprehension (e.g., differences in gaze behaviour between participant groups and sentence types), indicating that it was sufficiently sensitive to detect the main experimental effects. Nonetheless, webcam eye tracking showed a delay in the emergence of experimental effects (~500–800 ms) relative to lab-based eye tracking. This provides important empirical support that even very low–sampling-rate webcam recordings could capture meaningful psycholinguistic effects. Complementary evidence comes from Hutt et al. (2023), who used WebGazer to record gaze data during online reading and found that webcam-based eye movement features predicted comprehension errors and episodes of mind wandering. These findings show further promise for the use of low-sampling-rate webcam eye tracking to study reading under conditions that approximate natural reading.

Since it seems that the temporal resolution of webcams may, at least in theory, be sufficient for eye tracking during reading, the question is whether the quality of the spatial data obtained by webcam eye tracking is good enough to provide useful information for reading research. Spatial data quality can be quantified in two ways: Accuracy is the difference between the recorded gaze position and the true gaze position, while precision indicates whether the eye tracker gives consistent measurements. As screen sizes and viewing distances vary, accuracy is usually given as the average error in degrees of visual angle and precision is given as the root mean square of the sample-to-sample distances (S2S RMS), again in degrees of visual angle. For example, Angele et al. (2025) found that the average error for the EyeLink Portable Duo (a high-precision, laboratory-based eye tracker) was between 0.44 and 0.50 degrees of visual angle (using calibration points as the ground truth) and the S2S RMS was between 0.50 (at 250 Hz) and 0.11 degrees of visual angle (at 2,000 Hz).

In the last 10 years, a few online, browser-based eye-tracking software packages have become available. The first of these is WebGazer (Papoutsaki et al., 2016), which uses an open-source JavaScript algorithm to detect face, eyes, and eye features (such as pupil shape) in real time and continuously self-calibrates by assuming that the user is looking at the location of any mouse movements and clicks made. WebGazer is relatively straightforward to deploy by adding a few lines to the code of any website and, given its relative computational simplicity, works on most devices that have a modern browser supporting JavaScript, a webcam, and mouse or touch input. The authors claim that they achieve sufficient accuracy to obtain useful gaze position data, with an average error of 4.17º of visual angle compared with a laboratory-based eye tracker (Tobii EyeX). They give no information on precision. Even though this error seems quite large relative to a laboratory-based eye tracker, some very encouraging results have been found for the use of WebGazer in the visual world paradigm.

The visual world paradigm involves presenting participants with a display containing a number of objects (the “visual world”) while presenting a spoken sentence, without giving them any instructions except to listen to the sentence and look at the scene. Tracking participants’ eye movements in this task reveals that they tend to look at any object that is mentioned in the spoken sentence and that these gaze shifts occur as soon as it becomes clear that an object will be mentioned. For example, in Altman and Kamide’s (1999) study, participants shift their gaze from an image of a boy to an image of a cake as soon as they hear “eat” in “The boy will eat the cake”, as long as the cake is the only edible object in the scene (verb semantic constraint effect). This makes the visual world paradigm very useful for investigating the time course of spoken language processing (see Huettig et al., 2011, for a review).

What makes the visual world paradigm attractive for online eye tracking research is that the objects tend to be large and well separated. This makes it easy to determine which object a participant is looking at even if accuracy is relatively low. Slim and Hartsuiker (2023) successfully replicated a visual world experiment with bilingual participants conducted by Dijkgraaf et al. (2017), although they reported a smaller effect size than that found in the original experiments. Additionally, Slim and Hartsuiker (2023) found that the WebGazer-based fixation effects emerged approximately 300 ms later relative to the spoken sentence than in the lab-based study by Dijkgraaf et al. (2017). This onset delay suggests that webcam-based tracking can distort not only spatial estimates but also the temporal alignment of gaze relative to stimulus onset, and that such delays are unlikely to be consistent across studies or paradigms. Similarly, Prystauka et al. (2023) successfully replicated the verb semantic constraint effect first observed in the visual world paradigm by Altmann and Kamide (1999) and the lexical interference effect first observed by Kukona et al. (2014), where participants exhibit a small but consistent tendency to look at an object that is incompatible with the preceding verb they hear when a compatible colour word occurs (e.g., a black hat for the sentence “The grandfather will smoke the black pipe”). Prystauka et al. interpreted this as evidence that online eye tracking experiments are able to detect even relatively subtle effects, at least in the visual world paradigm.

In contrast, James et al. (2025) also attempted a replication of Altmann and Kamide’s (1999) results using WebGazer and failed to find evidence for the effect. This may be because their objects were relatively small and not always well separated (as in the original Altmann and Kamide study), as opposed to the four-quadrant design used by Prystauka et al. (2023), and highlights the accuracy issues that WebGazer has even in a design with relatively large stimuli. James et al. (2025) also tried to replicate the WebGazer experiment in the laboratory, using the built-in webcam of a MacBook laptop, and again failed to find evidence for the effect, suggesting that the issue is not due to the variability of participants’ devices and environments, but rather due to the inherent limitations of WebGazer. They did, however, succeed in replicating a number of other paradigms, including a visual world experiment reported by Ryskin et al. (2017), all using four-quadrant or two-halves designs. These limitations align with findings from a recent systematic evaluation of webcam-based eye-tracking algorithms, which highlighted that spatial drift, temporal latency, and browser-specific variability remain major obstacles to achieving laboratory-level precision (Thilderkvist & Dobslaw, 2024).

A number of other studies similarly find that online eye-tracking studies with designs analysing very large portions of the screen as areas of interest (as opposed to smaller, more detailed areas) tend to be successful when using WebGazer either on its own or integrated into an experiment platform such as Gorilla (Kandel & Snedeker, 2025; Slim et al., 2024; Steffan et al., 2024), even with young children or infants as participants.

Beyond these replication efforts, Bramlett and Wiener (2025) advanced the methodological foundations of web-based eye tracking by introducing an open-science workflow for conducting and analysing online visual world experiments. Using WebGazer and Gorilla, they demonstrated that systematic calibration, preprocessing, and mixed-effects modelling can enable reproducible and interpretable analyses of online eye-tracking data, thereby bridging the gap between feasibility studies and standardised research practice. Furthermore, Patterson et al. (2025) outline best-practice guidelines for webcam-based eye tracking, including calibration routines, data cleaning procedures, and transparent reporting.

Compared with the visual world paradigm, reading research requires higher precision, at least if the goal is to go beyond global measures such as the number of fixations on a passage. To put an error of 4.17º into perspective, one letter in 20 pt Courier New font, viewed at a distance of 60 cm on a 24-inch LCD monitor at a resolution of 1,920 × 1,080 pixels, subtends about 0.34º. This means that, with an error of 4.17º, the reported gaze location would be on average more than 12 letters away from the true location, making it very challenging to determine which word a participant is looking at and calculating accurate word-based fixation time measures. In comparison, the laboratory-based eye tracker, with an error of 0.5 degrees, would on average report a gaze location 1.4 letters away from the true gaze location, which, in most cases, is still inside the word on which the participant is fixating. As we describe below, this problem can be mitigated using a larger font size, but this limits the length of the stimuli that can be fit on the screen.

If we wish to achieve average accuracy of 1.4 letter spaces on an eye tracker with accuracy of 4.17º, each letter would have to subtend 3º of visual angle. If the visual angle subtended by a large screen is about 55º, we would only be able to fit 18 characters on a line, which is barely enough for three or four words. Additionally, the self-calibration mechanism used by WebGazer is not ideal for a reading paradigm, as reading does not require frequent mouse interaction—in fact, frequent use of the mouse may be indicative of a lack of focus on reading. To our knowledge, there has been only one attempt to collect data on eye movements during reading with WebGazer. The WebQAmGaze project (Ribeiro et al., 2024) involved the collection of 600 online eye movement recordings made using WebGazer while participants were reading paragraphs from existing lab-based eye movement corpora such as the Multilingual Eye-movement Corpus (MECO, Kuperman et al., 2025; Siegelman et al., 2022) in English, Spanish, German, and Turkish. The comparison of the WebGazer data with the original MECO data—which were obtained on high-precision eye trackers—showed some promising results, with clear correlations between the two datasets; however, the authors only present one analysis involving word properties (the word length effect), and, while the WebGazer data show a numerical tendency in the same direction as the MECO, the amount of noise in the data is substantial and may make it difficult to draw strong conclusions based on the WebGazer data alone. Additionally, the analysis is based on different sets of participants.

There have been several new online eye-tracking projects that have attempted to improve on the precision of WebGazer. Using customised software on a Google Pixel smartphone, Valliapan et al. (2020) claim to have achieved an average error of about 0.6–1.0 degrees of visual angle, which would make their system’s accuracy comparable to high-precision, lab-based eye trackers. While this is an exciting proof of concept showing what might be possible, the authors state that they are unable to release the software as it contains proprietary Google code, and, 6 years after the publication of the original article, no other study has used this software.

Taking a different approach, Saxena et al. (2023) used deep learning models to perform offline gaze detection on video recordings of participants’ faces that were collected in an online experiment. They report an average error of about 2.4 degrees of visual angle, which is still a significant improvement compared with WebGazer. Saxena et al.’s software and data are all available as open-source. However, the offline analysis approach raises the privacy concerns described above and, at the very least, requires specific consent from the participants for their faces to be recorded and the videos (which are personally identifiable information) to be stored on the researchers’ systems in order to be analysed.

Another attempt at improved software for online eye tracking comes from the Labvanced experimental platform (Finger et al., 2017). Similar to WebGazer, Labvanced performs all data processing on the participant’s machine, but it has a more sophisticated head and gaze model based on machine learning. While the use of Labvanced requires a paid licence and the code is closed-access, the platform is currently publicly available, and adding webcam eye tracking to an existing experiment is straightforward. To test the accuracy and precision of the Labvanced eye-tracking component, Kaduk et al. (2023) performed an experiment in which they recorded eye-tracking data simultaneously on an SR Research EyeLink 1000 Plus and using a webcam with the Labvanced software while participants were performing a variety of attention and eye-movement-related tasks. They claim average accuracy of 1.4º of error and precision of 1.1º, both of which are very impressive compared with WebGazer, especially given that the Labvanced platform works on most devices with a webcam. Kaduk et al. included a number of tasks in their study but did not include a reading task.

From the publicly available software solutions for online eye tracking reviewed above, Labvanced seems to be a promising one in terms of its reported accuracy and precision. Therefore, in the present study, we tested to what extent webcam eye tracking can be used to study eye movements during reading online. Similar to Kaduk et al. (2023), we directly compared the gaze position data obtained from Labvanced with the data from a high-precision SR Research EyeLink system using a co-registration set-up. Additionally, similar to Angele et al. (2025), we attempted to replicate a key benchmark effect from the reading literature using webcams—the word frequency effect (Godwin et al., 2025). By simultaneously examining the lexical frequency effect with both a webcam and a standard lab eye tracker, it was possible to test to what extent, if any, the two methods produce comparable results.

We report three experiments: Experiment 1 was a lab-based co-registration study using English sentences presented as 3–4 lines of text on a large lab monitor. Each sentence contained a target word frequency manipulation, and participants read the sentences while their eye movements were recorded by both an EyeLink 1000 Plus and the Labvanced system using an external webcam. An additional set of very short single-line sentences without any manipulation were included as a control. If the Labvanced system produces accurate and precise eye-movement data, we should see a high correlation between the EyeLink and the webcam results. Additionally, if the Labvanced system is accurate and precise enough to detect evidence of the cognitive processing of word properties in word-based fixation time measures, we should observe effects of word frequency in both the EyeLink and the webcam data. In Experiment 2, we conducted a replication in Spanish and further optimised the presentation of sentences so that they appear only on two lines (at the top and the bottom of the screen). In this experiment, we again used a lab-based set-up but with the stimuli presented on a laptop screen rather than a large computer monitor, which is more like the set-up that participants would use at home. Finally, in Experiment 3, we replicated Experiment 1 as a pure online experiment to test whether our findings would hold up in a remote, self-administered setting where the equipment and the environment were fully controlled by the participant.

## Experiment 1

### Method

#### Participants

Forty-two^1^ English speakers from the London UCL [University College London] area participated in return for a £10 payment (23 female). Participants’ average age was 24.7 years (*SD* = 7.6 years; range, 18–49 years). Participants reported normal or corrected-to-normal vision (with contact lenses) and no prior diagnosis of reading disorders or any other neurological disorders. Participants wearing glasses were screened out due to incompatibility with the webcam recording. The study was approved by the Research Ethics Committee chair at the Department of Experimental Psychology, UCL (no. EP_2024_01). Sample size was determined based on a previous study with the same sentence materials, which found reliable lexical frequency effects with 40 participants using an EyeLink 1000 eye tracker (Vasilev et al., 2019, Experiment 1). Due to the uncertainty of possible webcam effects, more precise planning was not possible.

#### Materials

The reading materials consisted of two sets of stimuli: 60 single sentences with a lexical frequency manipulation (henceforth, “Frequency corpus”) and 40 single-line sentences (henceforth, “Single-line corpus”). The presentation order of the two corpora was counterbalanced across participants. Because a large font size had to be used to offset the inherent error of the webcam system, the Frequency corpus sentences appeared on 3–4 lines of text across the whole screen. The single-line sentences all fitted on a single line of text and were used as a control to test how the webcam system performs in situations where tracking the *y*-position of the eye is not necessary.

##### Frequency corpus

The stimuli were adapted from Vasilev et al.’s (2019) Experiment 1 (see Table 1). They consisted of 60 sentences that included a target word, which was manipulated to be of either high or low lexical frequency (measured with the SUBTLEX-UK corpus; van Heuven et al., 2014). An example sentence is given below (target word is underlined for illustration purposes but appeared normally in the text):

**Table 1:** Psycholinguistic properties of the target words in Experiment 1 from the Frequency corpus

	High-frequency target words				Low-frequency target words			
Variable	Mean	*SD*	Min	Max	Mean	*SD*	Min	Max
Lexical frequency (Zipf)	5.12	0.290	4.69	5.84	3.32	0.376	1.84	4
Cloze predictability	0.006	0.02	0	0.095	0.007	0.036	0	0.238
Word length	5.38	1.04	3	7	5.38	1.04	3	7



The target words were an equal number of nouns and adjectives and differed significantly in lexical frequency, *t*(110.81) = 29.33, *p *< 0.001, but were matched on cloze predictability, *t*(91.71) = −0.299, *p* = 0.765, and word length (*p* = 1). The sentences were on average 11.73 words (*SD* = 1.53) or 65.2 characters long (*SD* = 6.96). The position of the target word in the sentence differed for each item but was never the first or last word (mean position = 6.21; *SD* = 1.65). The sentences were displayed on 3–4 lines of text; line breaks were created in such a way that the target word was never the first or last word on the line. Some of the original sentences were lightly edited (after the target word) to make them fit on the screen due to the larger letters. Each sentence was followed by a Yes/No comprehension question (e.g., “Does Mrs. Clark get along with everybody?”). The presentation of sentences across the two conditions was counterbalanced with a Latin square design, such that participants saw either the high- or low-frequency version of each sentence, but across participants, both versions were presented equally often.

##### Single-line corpus

The stimuli consisted of 40 very short sentences that were specifically written to fit on a single line of text (thus avoiding the need to track the *y*-position of the eye). They were 3–5 words long (*M* = 4.225; *SD* = 0.48 words) and contained 20.35 characters on average (*SD* = 0.863). Some examples are given below:

- The man was fed up.
- The cat saw the bird.
- The green vase broke.

Each sentence was followed by a Yes/No question (e.g., “Was the man pleased?”, “Did the cat see a dog?”).

#### Apparatus

Reference eye movements were recorded with an EyeLink 1000 Plus at 1,000 Hz (SR Research, Ontario, Canada). This was taken as the “ground truth” for comparing the accuracy of the webcam. However, as described above, the EyeLink 1000 Plus system has an average spatial error of 0.25–0.5° itself, so the accuracy is not absolute. Viewing was binocular, but only the right eye was recorded. Participants’ heads were stabilised with a chin-and-forehead rest. Data were recorded using a custom Python script (https://github.com/martin-vasilev/Eyelink_screen_recorder/). Calibration and validation of the EyeLink were performed using a nine-point grid at the start of the experiment. Participants were then recalibrated every 15 trials. The mean subject calibration error was 0.46° (*SD* = 0.10°; range, 0.27–0.65°).

Concurrent webcam eye movements were recorded with a Logitech StreamCam at 60 Hz using the webcam interface at labvanced.com (Kaduk et al., 2023). Due to a technical issue, the first three participants were recorded at a 30 Hz sampling rate. The webcam interface did not achieve the full sampling rate, and the effective sampling rate was 56.35 Hz on average (58.35 Hz if excluding the three participants recorded at 30 Hz). The webcam interface has reported average accuracy of 1.45° (*SD* = 0.76°) (Kaduk et al., 2023). The calibration procedure consisted of nine calibration screens—seven screens consisting of dot calibration grids (approx. 12 targets each) and two consisting of smooth pursuit screens (see Kaduk et al., 2023, for more details). The points during calibration are separated into a training set and a validation set; a model is then built on the training set to calculate the subsequent accuracy on the validation set (Caspar Goeke, June 2025, personal communication). The average validation error reported by the system was 0.81° (*SD* = 0.76°) for *X* coordinates and 0.45° (*SD* = 0.36°) for *Y* coordinates. However, due to the different methods of calculation, we advise against comparing those values with the calibration accuracy of the EyeLink system.

To synchronise the two eye-movement data streams, UNIX timestamps were sent to the EyeLink computer (Kaduk et al., 2023), which gave the same time reference point. This was done at the start of the experiment and then every five trials to resynchronise the internal clocks. The EyeLink data were downsampled offline to the effective sampling rate of the webcam for each participant. This was done by identifying the EyeLink sample that occurred at the UNIX time point when the image was first acquired from the webcam. The two samples were then combined into the same dataset for analysis. Note that all subsequent results and data processing steps reported in this paper are based on the downsampled EyeLink data.

**Fig. 1:**
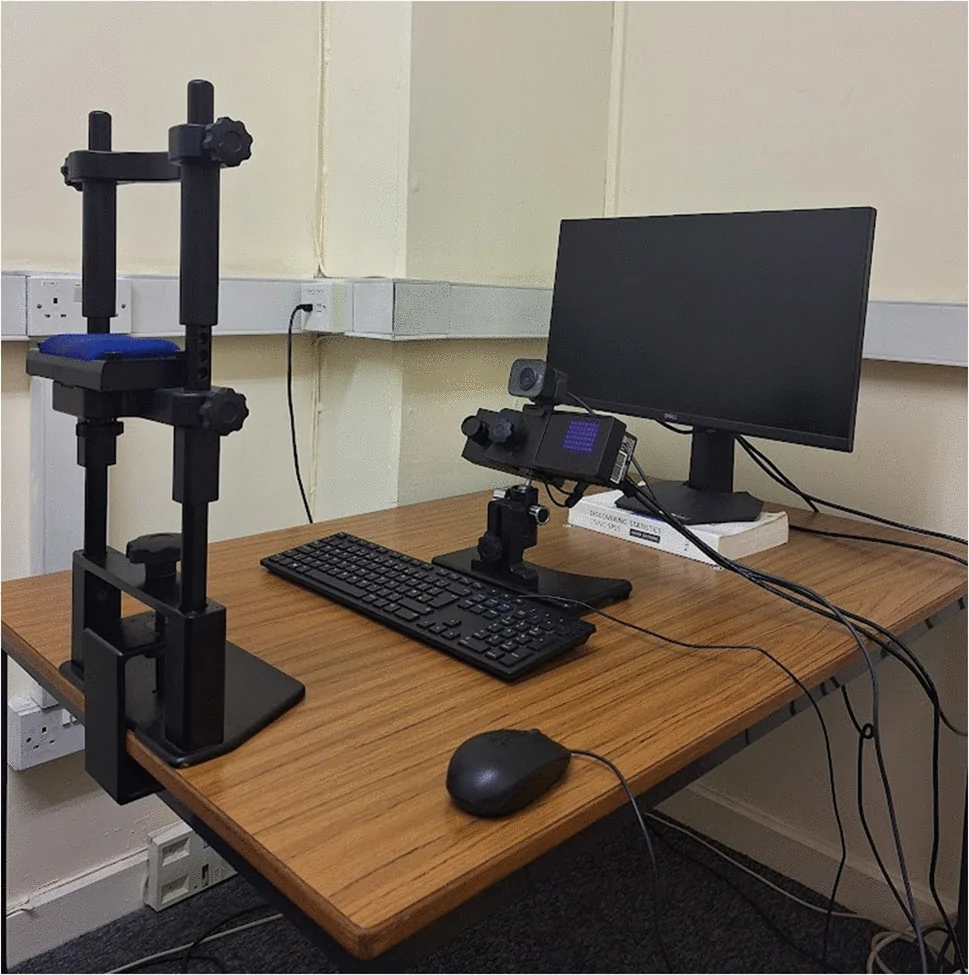
An illustration of the data acquisition set-up in Experiment 1

The experimental set-up is illustrated in Fig. 1. The stimuli were presented on a Windows 11 PC with a 24-inch LCD monitor (resolution, 1,920 × 1,080 pixels; refresh rate, 165 Hz; physical screen size, 53 cm × 30 cm). The distance between the eye and the EyeLink 1000 Plus was 55 cm, and the distance between the eye and the webcam was 50 cm. The Logitech StreamCam was mounted on top of the EyeLink such that it did not obstruct the camera, illuminator module, or block the participant’s view of the monitor. The eye-to-monitor distance was 82 cm. At this distance, each pixel subtended 0.0187° horizontally and 0.0192° vertically.

The sentences were formatted in a 60 pt. monospaced font that appeared as black text on a white background. The text was offset at 125 px horizontally and 150 px vertically. The text was double-spaced and aligned to the left. The width of each letter was 78 px (1.46°), matching the reported spatial accuracy of the webcam (1.45°; Kaduk et al., 2023).

#### Procedure

Participants were tested in a small windowless room illuminated by an overhead lamp (which was not in view of either camera). Participants provided informed written consent and were positioned on a chin and forehead rest (see Fig. 1). Once the webcam detected the position of the participants’ face, the chinrest was removed, and participants completed the webcam calibration. The chinrest was removed during this procedure because participants needed to shift their head position slightly during the calibration process (see Kaduk et al., 2023). Afterwards, participants were repositioned on the chinrest and were calibrated on the EyeLink eye tracker.^2^ Then the experiment commenced.

The study started with six practice trials, followed by the 100 experimental trials (60 in the Frequency corpus, 40 in the Single-line corpus). The sentences in the two corpora were blocked, but within each block, the sentences were presented in pseudo-random order. Each trial began with a fixation cross (82 × 82 pixels) placed at the first letter of the sentence. After the webcam detected the participant’s gaze on the area around the fixation cross, the sentence was presented on the screen. The experimenter manually presented the sentence with a keypress if the gaze-contingent presentation of the sentence was not triggered within a few seconds. Participants then read the sentence silently at their own pace and clicked the left button of the mouse to indicate they had finished reading. After each sentence, there was a True/False comprehension question, which participants answered with a mouse click. Targets to monitor drift were presented every fifth trial. For the EyeLink, a single drift check target was presented at the centre of the screen, which did not affect the calibration model. For the webcam interface, a seven-point drift correction grid was presented. This grid updated gaze coordinate predictions by cross-validating the systematic error across all seven points (Kaduk et al., 2023). The EyeLink was recalibrated every 15 trials. For the webcam, the seven-point drift correction grid was also used to update the initial calibration model every 15 trials. The study took about 45 min to complete.

#### Data analysis

The raw data were first cleaned by removing samples occurring outside the screen area (2.09%), as well as any samples that occurred during a blink (5.09%). For the EyeLink, blinks were defined as any samples where the pupil size was 0. For the webcam interface, a confidence rating (0–1) indicated whether the eyelid was open. We selected only cases where the rating was > 0.

To assess eye-tracking consistency between the two signals, we computed Pearson’s correlation coefficient between the webcam and the downsampled EyeLink gaze coordinates separately for the horizontal (*x*) and vertical (*y*) dimensions. This approach was inspired by Kaduk et al. (2023), who reported high overall correlations between the two signals. Furthermore, to estimate the relative error across screen regions, we divided the display into a 3 × 3 grid based on the monitor resolution. Each gaze sample was assigned to one of nine equally sized sections (Sections 1–9 from top left to bottom right), and the difference between the webcam and EyeLink gaze locations was calculated for each region.

To analyse reading behaviour, the raw eye samples from both systems were parsed into fixations using Engbert and Kliegl’s (2003) algorithm, as implemented in the “saccades” R package (von der Malsburg, 2019). We used the default λ = 6 parameter for saccade detection sensitivity and a three-sample moving window smoothing of gaze coordinates to reduce noise. This algorithm has previously been shown to be sensitive in low-sampling-resolution applications for detecting the lexical frequency effect (Angele et al., 2025). The algorithm identified 43,796 fixations in the EyeLink data (33,422 in the Frequency corpus; 10,374 in the Single-line corpus) and 32,868 fixations in the webcam data (25,176 in the Frequency corpus; 7,692 in the Single-line corpus). The webcam data resulted in fewer, but longer, fixation durations detected by the algorithm (see Appendix Fig. 9).

Fixations shorter than 80 ms that were within one-character distance of a temporally adjacent fixation were merged into that adjacent fixation (Frequency corpus, 0.38% [webcam], 1.76% [EyeLink]; Single-line corpus, 0.56% [webcam], 2.32% [EyeLink]); any remaining fixations under 80 ms were discarded as outliers (Frequency corpus, 3.46% [webcam], 16.17% [EyeLink]; Single-line corpus, 3.94% [webcam], 15.82% [EyeLink]).^3^ Additionally, fixations longer than 1,000 ms were also discarded as outliers (Frequency corpus, 2.93% [webcam], 0.47% [EyeLink]; Single-line corpus, 4.22% [webcam], 0.52% [EyeLink]). Downsampling was performed prior to fixation detection and data cleaning so that both signals were processed at a comparable temporal resolution. Note that all EyeLink processing steps are based on the downsampled data.

From the raw fixation data, we computed the following reading measures: *first fixation duration* (FFD; the duration of the first fixation on a word during first-pass reading); *single fixation duration* (SFD; the duration of a fixation on a word when it is fixated exactly once during first-pass reading), *gaze duration* (GD; the sum of all fixation durations on a word during first-pass reading), *total viewing time* (TVT; the sum of all fixations on a word, including when a word is re-fixated during second-pass reading), and *first-pass skipping probability* (the probability of skipping the word during first-pass reading). We trimmed durations above 2,000 ms for GD and 3,000 ms for TVT as outliers (Frequency corpus, 0.22% [webcam], 0.09 % [EyeLink]; Single-line corpus, 4.02% [webcam], 0.59% [EyeLink]).

We analysed the data for the Frequency corpus by fitting Bayesian (generalised) linear mixed models using the brms package (Bürkner, 2017, 2018, 2021) in R^4^ These models included the word frequency condition (low, represented as −0.5, versus high, represented as 0.5) as well as the eye tracker used (with the EyeLink represented as −0.5 and the webcam represented as 0.5). We included the maximum random-effects structure (i.e., random intercepts, random slopes for Frequency and Tracker, and their interaction over both subjects and items, Barr et al., 2013). We used the Gaussian distribution to model log-transformed fixation times and the Bernoulli family to model skipping probabilities. For all models, the default priors suggested by brms were used, except for the slope coefficients, which were given weakly informative priors of *b ~Normal(0, 1)* to rule out implausibly large effects.

Each model was fitted using four chains with 5,000 iterations each, with 1,000 warmup iterations. The models converged successfully (all R-hats = 1.00). We report the estimates (*b*) and the 95% Bayesian credible intervals (95% CrIs) based on the posterior distribution of each model parameter and indicate whether 0 is excluded from the 95% CrI. The exclusion of 0 from the 95% CrI was the main method of inference used to determine the presence of an effect. Additionally, we report the proportion of the posterior on the same side of 0 [P(*b*>0) or P(*b*<0)] and its ratio to the opposite-side proportion as a directional evidence ratio (ER_b>0_ or ER_b<0_), which can be interpreted as a Bayes factor for the directional hypothesis. Finally, we report Bayes factors (Jeffreys, 1961) for the point-null hypothesis (*b* = 0), calculated using the Savage–Dickey density ratio method (Dickey & Lientz, 1970; Morey et al., 2011). It is important to note that each of these statistics is a valid way to summarise the evidence provided by the data, but, for simplicity, we will focus on the 95% CI and whether it excludes 0 in our verbal description of the results.

### Results and discussion

Participants’ mean comprehension accuracy was 96.3% in the Frequency corpus (*SD* = 19.0%; range, 73.3–100%) and 97.1% in the Single-line corpus (*SD* = 16.9%; range, 85–100%). This shows that participants were reading for comprehension. The average total sentence reading times were 3,708 ms for the Frequency corpus (*SD* = 2,026 ms) and 1,799 ms for the Single-line corpus (*SD* = 1,043 ms). Because total sentence reading time is determined by the participant’s manual response, and both eye trackers recorded the same trial simultaneously, this duration was necessarily identical for the webcam and EyeLink data within each trial, confirming that the substantially longer timed measures observed for the webcam (Table 2) reflect differences in fixation detection rather than differences in overall time on task.

**Table 2:** Mean first fixation duration (FFD), single fixation duration (SFD), gaze duration (GD), total viewing time (TVT), and first-pass skipping probability on target words in Experiment 1, grouped by eye tracker and target word frequency condition

		FFD		SFD		GD		TVT		Skipping probability	
Tracker	Frequency	Mean	*SD*	Mean	*SD*	Mean	*SD*	Mean	*SD*	Mean	*SD*
Webcam	Low	281	173	287	174	351	237	559	390	0.53	0.50
Webcam	High	265	159	273	169	330	210	522	399	0.54	0.50
EyeLink	Low	190	133	197	146	236	163	342	253	0.18	0.38
EyeLink	High	176	124	183	135	207	142	299	230	0.22	0.41

All means and standard deviations are reported in milliseconds (ms), except for skipping probability, which is reported as probability

#### Comparison of raw sample positions

**Fig. 2:**
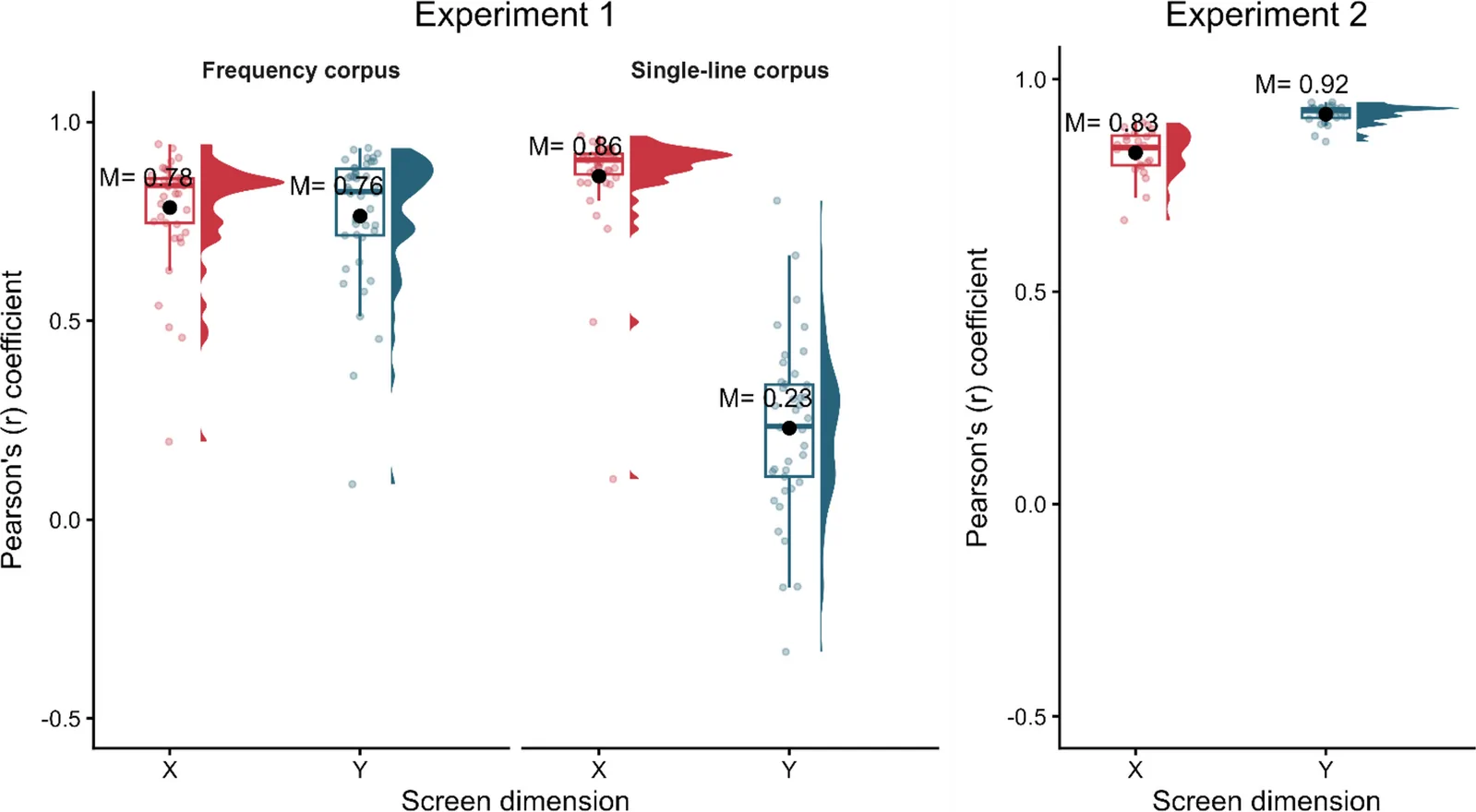
Pearson’s *r* correlations between the EyeLink and webcam gaze sample position in Experiments 1 and 2, calculated separately for the *x*- and *y*-dimension. Each dot represents the average correlation for a participant in the study. The black dot represents the average across all participants

To measure the relative consistency in the two signals, the two eye-tracker samples were correlated separately for the *x* and *y* dimensions of the screen (see Fig. 2). The average correlations were *r* = 0.78 (*x*-dimension) and *r* = 0.76 (*y*-dimension) for the Frequency corpus. These values align closely with the *r* = 0.83 (*X*) and *r* = 0.84 (*Y*) reported by Kaduk et al. (2023) in a free-viewing task. The average correlations were *r* = 0.86 (*x*-dimension) and *r* = 0.23 (*y*-dimension) for the Single-line corpus. The low correlation for the *Y*-axis likely shows that the webcam is less able to capture small deviations in the *y* position of the eye in a task where vertical eye movements are not needed.

The agreement between the two systems was further explored with Bland–Altman (1999) plots. These are often used for comparing a new measurement system against an existing “gold” standard. The difference in raw eye positions between the two systems is plotted against their mean value. If the new system shows no error or bias, the differences will be distributed around 0 and exhibit a horizontal slope, with 95% of the values falling within the confidence limits. If the slope of the scatterplot is not 0, this shows a “proportional bias”—i.e., the difference between two systems changes with the magnitude of measurement (Ludbrook, 2010).

**Fig. 3:**
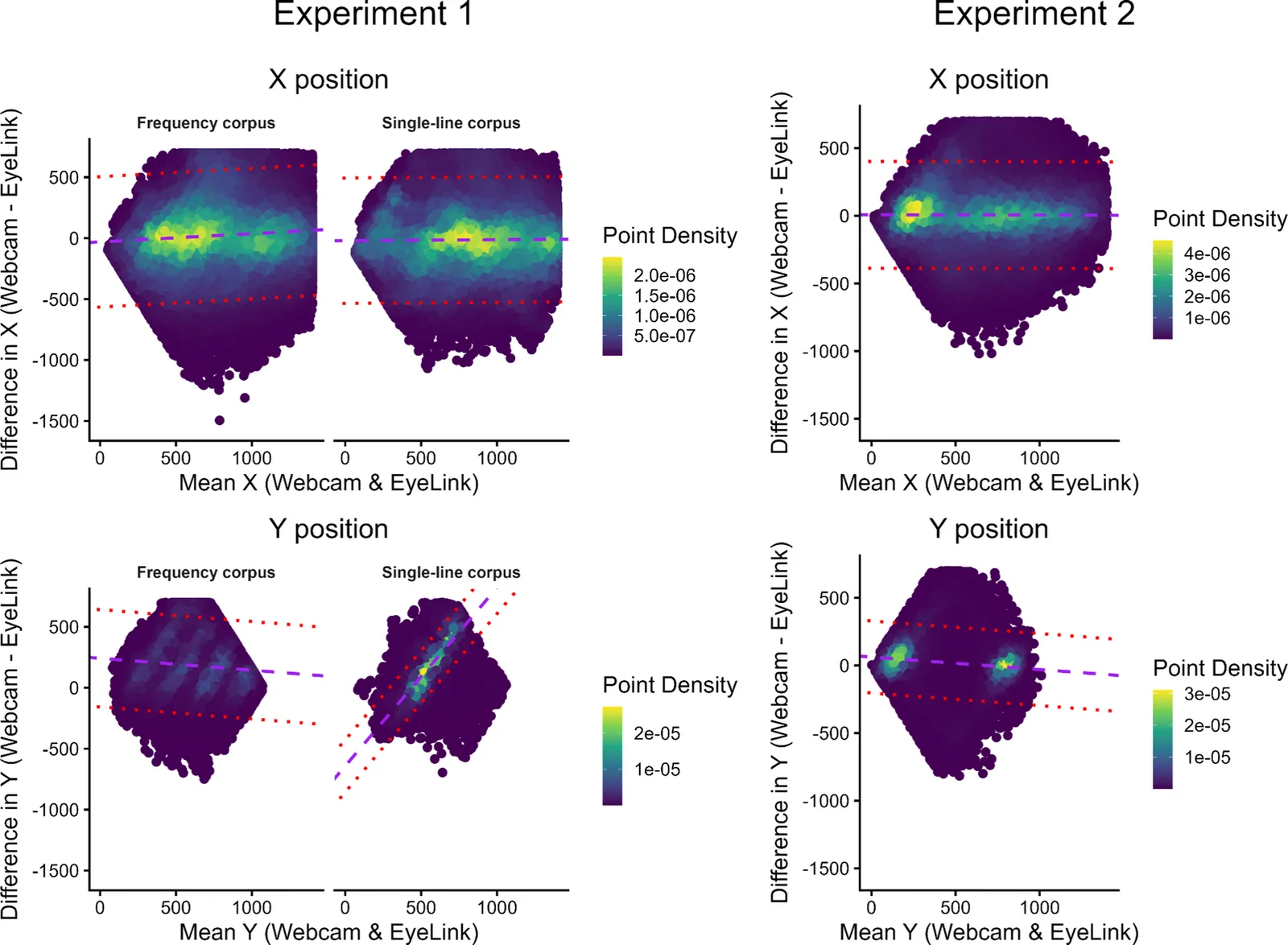
Bland–Altman (1999) plots showing the difference between the webcam and EyeLink raw eye positions for the *X*-axis (top row) and *Y*-axis (bottom row), plotted against the mean value for both cameras. In the absence of a systematic measurement bias, the differences should be distributed around 0, and the scatterplot should exhibit a horizontal slope. Dotted lines indicate the linear regression slope and 95% CI limits. The point density gradient shows the concentration of data points, with warmer colours indicating more observations in this location. Data from Experiment 1 are shown on the left, and data from Experiment 2 are shown on the right

As Fig. 3 shows, the webcam and EyeLink agreed well on estimating the *X* position of the eye, with little evidence of systematic bias. However, the confidence limits were very wide, indicating considerable uncertainty in the measurement. On the other hand, the *Y* position of the eye was consistently overestimated on the Frequency corpus. For the Single-line corpus, the opposite pattern was found—the webcam consistently underestimated low *Y* positions and overestimated high *Y* positions. As the Single-line corpus did not require participants to move their eyes vertically, this suggests that the webcam may not differentiate well between small fluctuations in the *Y* position of the eye.

**Fig. 4:**
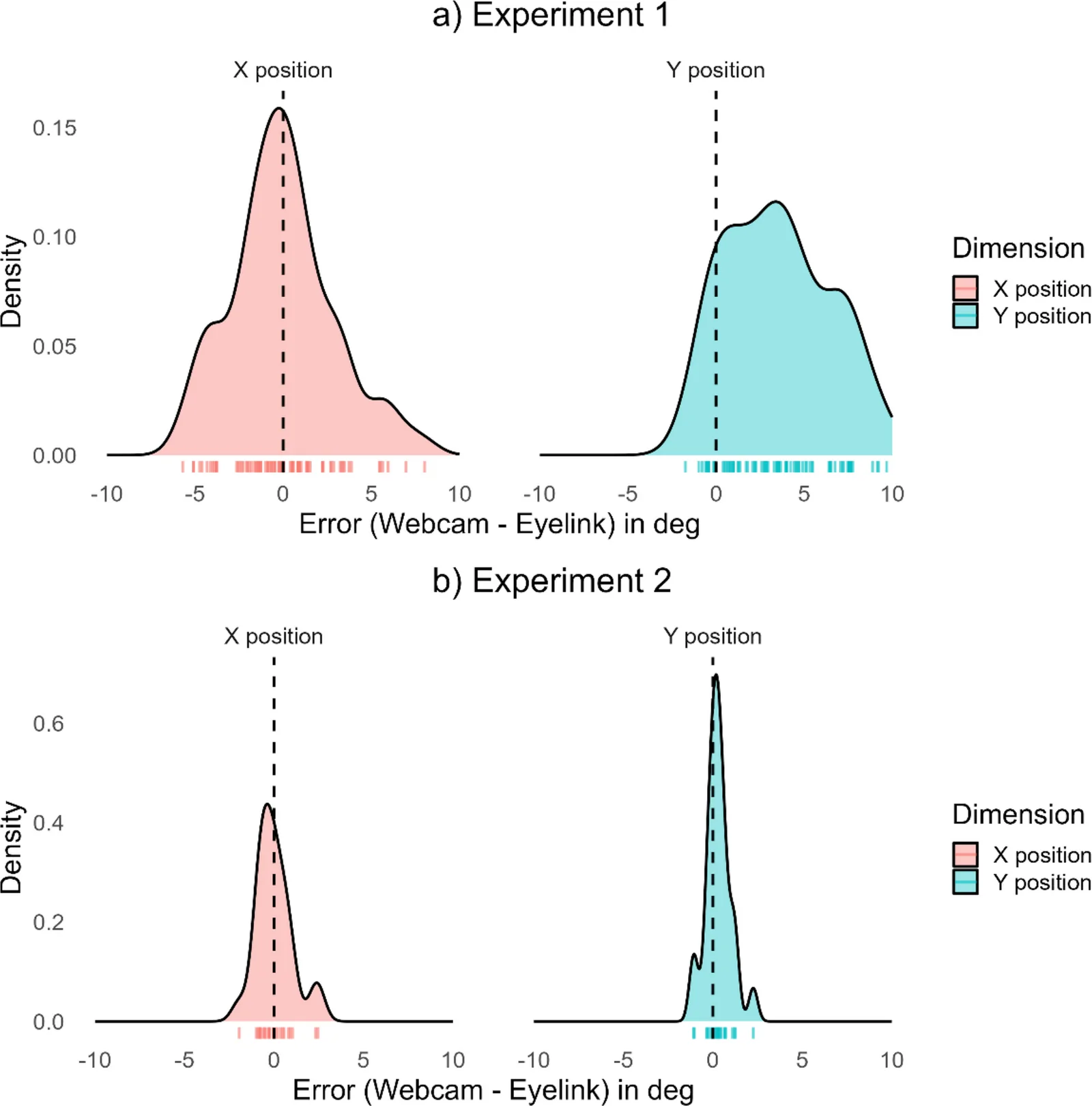
Average positional difference between the webcam and EyeLink recordings in Experiment 1 (**a**) and Experiment 2 (**b**), expressed in degrees of visual angle. The data were aggregated over subjects before plotting. In Experiment 1, the data were collapsed across the Frequency and Single-line corpora because the results were very similar. Negative values indicate an underestimation of the EyeLink gaze position by the webcam. A vertical dotted line at 0 is plotted to aid interpretation

**Fig. 5:**
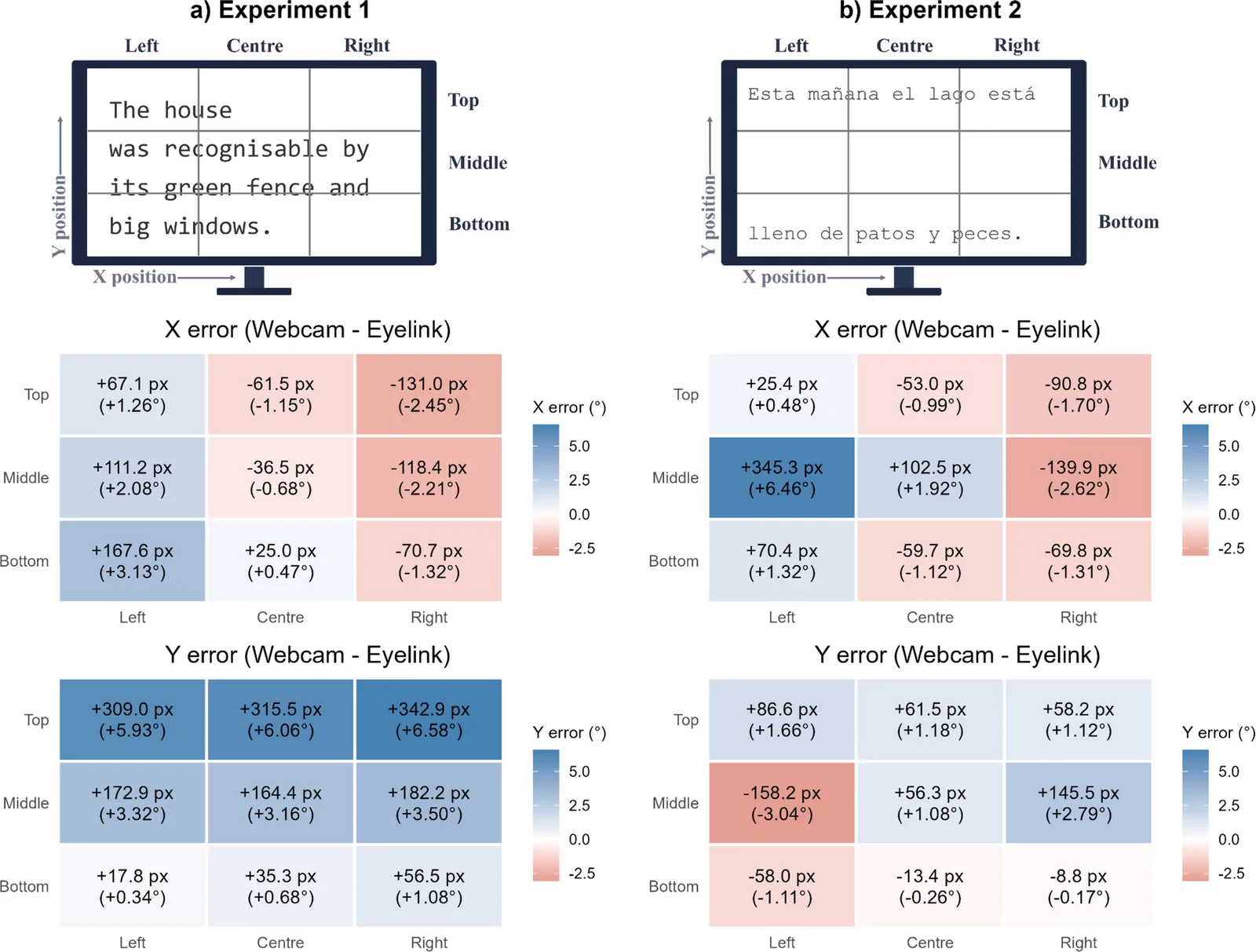
Mean positional error of webcam relative to EyeLink by screen section in Experiment 1 (**a**) and Experiment 2 (**b**), shown separately for horizontal (middle row) and vertical (bottom row) error. Experiment 1 shows all data (from both corpora). The display area was divided into a 3 × 3 grid, and gaze samples were assigned to sections according to their EyeLink coordinates. The top row shows a schematic of the 3 × 3 grid superimposed on the monitor, with an example stimulus sentence in the background. Tile colour indicates error direction (red for negative errors and blue for positive errors), with gradient showing the error magnitude. Labels inside each section indicate signed error values (in pixels and degrees). Positive errors indicate that the webcam overestimates the EyeLink gaze position, and negative errors indicate that it underestimates it

The average magnitude of the webcam error relative to the EyeLink was 0.25° (*SD* = 5.08°) for the *x*-dimension and 3.50° (*SD* = 3.83°) for the y-dimension (see Fig. 4a). This shows that the webcam estimated the average *x* position with little bias, though there was considerable variance around the mean. The *y* position was consistently overestimated by the webcam; there was a similar (though smaller) spread of values around the mean. We also compared the magnitude of the error based on the screen area where the samples occurred (separated into nine rectangles in a 3 × 3 grid). As Fig. 5a shows, the *x* position was recorded with the least amount of bias at the centre of the screen. *X* positions were overestimated on the left side of the screen and underestimated on the right side of the screen. However, across the whole screen area, horizontal errors largely cancelled each other out. On the other hand, the *y* position was consistently overestimated, with error magnitudes largest at the top third of the screen and smallest at the bottom third of the screen.

#### Analysis of the target word lexical frequency effect

We now turn to analysing fixation durations on the target word to check for evidence of the lexical frequency effect. Table 2 shows the means and standard deviations for the four fixation time measures and skipping probability on target words in Experiment 1, while Table 3 shows the corresponding linear mixed-effect model estimates.

**Table 3:** Bayesian linear mixed-effects models showing the target word lexical frequency effect of the categorical (high vs low) target word frequency manipulation in Experiment 1. The results show coefficient estimates and 95% credible intervals (in square brackets)

	FFD	SFD	GD	TVT	Skipping probability
Intercept	**5.301**	**5.318**	**5.469**	**5.821**	**−0.645**
	[**5.203, 5.400**]	[**5.212, 5.427**]	[**5.368, 5.570**]	[**5.711, 5.933**]	**[−1.064, −0.238]**
Frequency	**0.057**	**0.055**	**0.085**	**0.111**	−0.198
	[**0.018, 0.097**]	[**0.012, 0.098**]	[**0.039, 0.130**]	[**0.057, 0.165**]	**[**−0.407, 0.005**]**
Tracker	**0.412**	**0.409**	**0.431**	**0.553**	**1.981**
	[**0.333, 0.490**]	[**0.321, 0.496**]	[**0.344, 0.517**]	[**0.442, 0.667**]	**[1.360, 2.579]**
Frequency × Tracker	−0.037	−0.033	−0.082	−0.064	0.232
	[−0.109, 0.033]	[−0.112, 0.048]	[−0.172, 0.009]	[−0.160, 0.029]	[−0.118, 0.575]

Effects for which the 95% CrI excludes 0 are highlighted in bold. *FFD* first fixation duration; *SFD* single fixation duration; *GD* gaze duration; *TVT* total viewing time

Overall, low-frequency words elicited longer fixation times than high-frequency words across both devices, with the 95% CrI excluding 0 (FFD, b=0.06, 95% CrI $\left[0.02,\hspace{0.25em}0.10\right]$, $P\left(b>0\right)=1.00$, $E{R}_{b>0}=389.24$, $B{F}_{10}=1.14$; SFD, b=0.05, 95% CrI $\left[0.01,\hspace{0.25em}0.10\right]$, $P\left(b>0\right)=0.99$, $E{R}_{b>0}=151.38.$, $B{F}_{10}=0.47$; GD, b=0.09, 95% CrI $\left[0.04,\hspace{0.25em}0.13\right]$, $P\left(b>0\right)=1.00$, $E{R}_{b>0}>\mathrm{1,000}$, $B{F}_{10}=10.35$; TVT, b=0.11, 95% CrI $\left[0.06,\hspace{0.25em}0.17\right]$, $P\left(b>0\right)=1.00$, $E{R}_{b>0}>\mathrm{1,000}$, $B{F}_{10}=34.41$). Low-frequency words led to lower skipping probability, though the credible interval narrowly included 0 (b=-0.20, 95% CrI $\left[-0.41,\hspace{0.25em}0.0053\right]$, $P\left(b<0\right)=0.97$, $E{R}_{b<0}>34.71$, $B{F}_{10}=0.63$). Fixation times were also longer with the webcam than with the EyeLink and skipping rates were similarly higher (FFD, b=0.41, 95% CrI $\left[0.33,\hspace{0.25em}0.49\right]$, $P\left(b>0\right)=1.00$, $E{R}_{b>0}>\mathrm{1,000}$, $B{F}_{10}>\mathrm{1,000}$; SFD, b=0.41, 95% CrI $\left[0.32,\hspace{0.25em}0.50\right]$, $P\left(b>0\right)=1.00$, $E{R}_{b>0}>\mathrm{1,000}$, $B{F}_{10}>\mathrm{1,000}$; GD, b=0.43, 95% CrI $\left[0.34,\hspace{0.25em}0.52\right]$, $P\left(b>0\right)=1.00$, $E{R}_{b>0}>\mathrm{1,000}$, $B{F}_{10}>\mathrm{1,000}$; TVT, b=0.55, 95% CrI $\left[0.44,\hspace{0.25em}0.67\right]$, $P\left(b>0\right)=1.00$, $E{R}_{b>0}>\mathrm{1,000}$, $B{F}_{10}>\mathrm{1,000}$; skipping, b=1.98, 95% CrI $\left[1.36,\hspace{0.25em}2.58\right]$, $P\left(b>0\right)=1.00$, $E{R}_{b>0}>\mathrm{1,000}$, $B{F}_{10}>\mathrm{1,000}$). This may be due to the higher spatial resolution of the EyeLink system, which enabled the algorithm to detect shorter fixations that may have been missed in the webcam data due to its greater variance in gaze positions. Importantly, the frequency × tracker interaction was not credibly different from 0 (FFD, b=-0.04, 95% CrI $\left[-0.11,\hspace{0.25em}0.03\right]$, $P\left(b<0\right)=0.86$, $E{R}_{b<0}=5.90$, $B{F}_{10}=0.06$; SFD, b=-0.03, 95% CrI $\left[-0.11,\hspace{0.25em}0.05\right]$, $P\left(b<0\right)=0.79$, $E{R}_{b<0}=3.85$, $B{F}_{10}=0.06$; GD, b=-0.08, 95% CrI $\left[-0.17,\hspace{0.25em}0.009\right]$, $P\left(b<0\right)=0.96$, $E{R}_{b<0}=24.68$, $B{F}_{10}=0.22$; TVT, b=-0.06, 95% CrI $\left[-0.16,\hspace{0.25em}0.03\right]$, $P\left(b<0\right)=0.91$, $E{R}_{b<0}=10.21$, $B{F}_{10}=0.12$; skipping, b=0.23, 95% CrI $\left[-0.12,\hspace{0.25em}0.58\right]$, $P\left(b>0\right)=0.91$, $E{R}_{b>0}=9.63$, $B{F}_{10}=0.42$), indicating that the frequency effect was comparable across devices.

The absence of an interaction between frequency and tracker is not in itself positive evidence for a frequency effect in the webcam data. We calculated posterior distributions for the estimated marginal means of the difference between low- and high-frequency target words and found that the 95% credible intervals for these differences in the webcam data all included 0, while the credible intervals for the EyeLink data all excluded 0 in FFD (webcam, *M* = 0.04, 95% CrI [−0.02, 0.10]; EyeLink, *M* = 0.08, 95% CrI [0.04, 0.12]), SFD (webcam, *M* = 0.04, 95% CrI [−0.03, 0.11]; EyeLink, *M* = 0.07, 95% CrI [0.03, 0.12]), GD (webcam, *M* = 0.04, 95% CrI [−0.03, 0.12]; EyeLink, *M* = 0.13, 95% CrI [0.08, 0.17]), skipping (webcam, *M* = −0.08, 95% CrI [−0.36, 0.19]; EyeLink, *M* = −0.31, 95% CrI [−0.58, −0.04]), and TVT (webcam, *M* = 0.08, 95% CrI [−0.00, 0.16]; EyeLink, *M* = 0.14, 95% CrI [0.08, 0.20]), although the CrI for TVT included 0 very narrowly for the webcam data. This indicates that, while the webcam data are consistent with a frequency effect of a similar magnitude to that observed with the EyeLink, the evidence is not sufficiently strong to confirm the presence of a frequency effect based on the webcam data alone using the criterion of the 95% credible interval excluding 0. We assume that this is due to the higher amount of noise in the webcam data.

#### Analysis of corpus lexical frequency

By including all words in every sentence in the analysis, rather than only the target words, we can increase the number of observations. Of course, this comes at the cost of increased variability due to differences in word length, predictability, and other lexical and contextual factors, but overall, this approach is likely to have a better chance of detecting the word frequency effect. Table 4 shows the results of these “corpus-style” Bayesian linear mixed model analyses with Zipf frequency as a continuous predictor, tracker as a two-level factor (as in the target word analysis), and the interaction between the two predictors for the four fixation time measures on all words in the sentences (except for the first and last word in each sentence) in Experiment 1. Note that this analysis contains the combined items from both the Frequency corpus and the Single-line corpus (100 items in total).

**Table 4:** Bayesian linear mixed-effects models showing the corpus lexical frequency effect of continuous Zipf word frequency across all words (except for the first and last in each sentence) in Experiment 1. The results show coefficient estimates and 95% credible intervals (in square brackets)

	FFD	SFD	GD	TVT	Skipping probability
Intercept	**5.252**	**5.257**	**5.363**	**5.594**	**0.396**
	[**5.167, 5.341**]	[**5.174, 5.344**]	[**5.281, 5.448**]	[**5.512, 5.672**]	[**0.135, 0.659**]
Zipf frequency	**−0.037**	**−0.044**	**−0.079**	**−0.12**	**0.485**
	[**−0.046, −0.028**]	[**−0.053, −0.034**]	[**−0.090, −0.068**]	[**−0.135, −0.104**]	[**0.409, 0.560**]
Tracker	**0.411**	**0.409**	**0.452**	**0.547**	**1.179**
	[**0.349, 0.473**]	[**0.345, 0.473**]	[**0.375, 0.529**]	[**0.471, 0.620**]	[**0.894, 1.472**]
Zipf Frequency × Tracker	**0.020**	**0.023**	**0.019**	**0.017**	**−0.536**
	[**0.010, 0.030**]	[**0.012, 0.033**]	[**0.008, 0.031**]	**[0.005, 0.030]**	**[−0.602, −0.471]**

Effects for which the 95% CrI excludes 0 are highlighted in bold. *FFD* first fixation duration; *SFD* single fixation duration; *GD* gaze duration; *TVT* total viewing time

As expected, we observed a frequency effect in that a higher Zipf frequency (i.e., more frequent words) was associated with shorter fixation times and higher skipping probability across all measures (FFD, b=-0.04, 95% CrI $\left[-0.05,\hspace{0.25em}-0.03\right]$, $P\left(b<0\right)=1.00$, $E{R}_{b<0}>\mathrm{1,000}$, $B{F}_{10}>\mathrm{1,000}$; SFD, b=-0.04, 95% CrI $\left[-0.05,\hspace{0.25em}-0.03\right]$, $P\left(b<0\right)=1.00$, $E{R}_{b<0}>\mathrm{1,000}$, $B{F}_{10}>\mathrm{1,000}$; GD, b=-0.08, 95% CrI $\left[-0.09,\hspace{0.25em}-0.07\right]$, $P\left(b<0\right)=1.00$, $E{R}_{b<0}>\mathrm{1,000}$, $B{F}_{10}>\mathrm{1,000}$; TVT, b=-0.12, 95% CrI $\left[-0.13,\hspace{0.25em}-0.10\right]$, $P\left(b<0\right)=1.00$, $E{R}_{b<0}>\mathrm{1,000}$, $B{F}_{10}>\mathrm{1,000}$; skipping, b=0.49, 95% CrI $\left[0.41,\hspace{0.25em}0.56\right]$, $P\left(b>0\right)=1.00$, $E{R}_{b>0}>\mathrm{1,000}$, $B{F}_{10}>\mathrm{1,000}$). We again observed longer mean fixation times and higher skipping probability with the webcam than with the EyeLink (FFD, b=0.41, 95% CrI $\left[0.35,\hspace{0.25em}0.47\right]$, $P\left(b>0\right)=1.00$, $E{R}_{b>0}>\mathrm{1,000}$, $B{F}_{10}>\mathrm{1,000}$; SFD, b=0.41, 95% CrI $\left[0.34,\hspace{0.25em}0.47\right]$, $P\left(b>0\right)=1.00$, $E{R}_{b>0}>\mathrm{1,000}$, $B{F}_{10}>\mathrm{1,000}$; GD, b=0.45, 95% CrI $\left[0.37,\hspace{0.25em}0.53\right]$, $P\left(b>0\right)=1.00$, $E{R}_{b>0}>\mathrm{1,000}$, $B{F}_{10}>\mathrm{1,000}$; TVT, b=0.55, 95% CrI $\left[0.47,\hspace{0.25em}0.62\right]$, $P\left(b>0\right)=1.00$, $E{R}_{b>0}>\mathrm{1,000}$, $B{F}_{10}>\mathrm{1,000}$; skipping, b=1.18, 95% CrI $\left[0.89,\hspace{0.25em}1.47\right]$, $P\left(b>0\right)=1.00$, $E{R}_{b>0}>\mathrm{1,000}$, $B{F}_{10}>\mathrm{1,000}$).

There was a small but credible frequency × tracker interaction in FFD (b=0.02, 95% CrI $\left[0.01,\hspace{0.25em}0.03\right]$, $P\left(b>0\right)=1.00$, $E{R}_{b>0}>\mathrm{1,000}$, $B{F}_{10}=90.41$), SFD (b=0.02, 95% CrI $\left[0.01,\hspace{0.25em}0.03\right]$, $P\left(b>0\right)=1.00$, $E{R}_{b>0}>\mathrm{1,000}$, $B{F}_{10}=\mathrm{1,000}$), GD (b=0.02, 95% CrI $\left[0.008,\hspace{0.25em}0.03\right]$, $P\left(b>0\right)=0.99$, $E{R}_{b>0}=\mathrm{1,000}$, $B{F}_{10}=1.24$), TVT (b=0.02, 95% CrI $\left[0.004,\hspace{0.25em}0.03\right]$, $P\left(b>0\right)=1.00$, $E{R}_{b>0}=224.35$, $B{F}_{10}=0.21$), and skipping probability (b=-0.54, 95% CrI $\left[-0.60,\hspace{0.25em}-0.47\right]$, $P\left(b<0\right)=1.00$, $E{R}_{b<0}=\mathrm{1,000}$, $B{F}_{10}>\mathrm{1,000}$). This interaction indicates that the frequency effect was stronger in the EyeLink data than the webcam data. To show the marginal effect of Zipf frequency within each device directly, we summarised the slope of the frequency effect using *emtrends* from the *emmeans* package. We found consistent evidence for the frequency effect in all measures for both the EyeLink and the webcam (FFD: EyeLink, *M* = −0.05, 95% CrI [−0.06, −0.04]; webcam, *M* = −0.03, 95% CrI [−0.04, −0.02]; SFD: EyeLink, *M* = −0.05, 95% CrI [−0.06, −0.04]; webcam, *M* = −0.03, 95% CrI [−0.04, −0.02]; GD: EyeLink, *M* = −0.09, 95% CrI [−0.10, −0.08]; webcam, *M* = −0.07, 95% CrI [−0.08, −0.06]; TVT: EyeLink, *M* = −0.13, 95% CrI [−0.14, −0.11]; webcam, *M* = −0.11, 95% CrI [−0.13, −0.09]; skipping: EyeLink, *M* = 0.75, 95% CrI [0.67, 0.83]; webcam, *M* = 0.22, 95% CrI [0.13, 0.30). Overall, this demonstrates that, when there are sufficient data, the corpus frequency effect can be detected even in the noisier webcam data.

#### Summary and conclusions

The results of Experiment 1 can be summarised as follows. First, the time series correlations of the webcam and EyeLink data showed a strong association, replicating Kaduk et al.’s (2023) results in a free-viewing task. Second, the webcam gaze coordinates generally showed much larger variation than the EyeLink gaze coordinates. While little systematic bias was found in estimating *x* positions, *y* positions were generally overestimated for the Frequency corpus (multi-line reading). Third, reliable lexical frequency effects on the target word were found only for the EyeLink data. The effect in the webcam data was numerically similar, though the 95% CrIs all included 0, suggesting that the effect is not statistically reliable. To boost statistical power, we conducted a corpus frequency analysis of all words in the sentence (instead of just the target word). This analysis revealed reliable corpus frequency effects in the webcam data. This suggests that corpus frequency effects can be detected with a larger number of observations.

Overall, these results show promise in using webcam eye tracking for reading research. However, they also demonstrate some challenges, such as the considerable additional noise in estimating gaze coordinates and systematic errors in tracking *y*-positions. If webcam eye tracking is to be practically useful, some form of multiline reading is necessary to ensure that the stimuli can fit on the screen, given that letters need to be much larger to counter the inherent error in webcam tracking. The current study presented the Frequency corpus items over 3–4 lines of text; however, using two lines of text may reduce the vertical error associated with mapping fixations to the text. In Experiment 2, we conducted a conceptual replication of the study in Spanish, using sentences with a lexical frequency manipulation spanning 2 lines of text. The study was also conducted on a laptop (rather than a lab monitor), which is closer to the set-up that participants would have at home.

## Experiment 2

### Method

#### Participants

Twenty-four native Spanish speakers (15 female) from Universidad Nebrija participated in the study in exchange for payment of €6 for a 30-min experiment. Their average age was 26.4 years (*SD* = 3.4; range, 21–31 years). All participants reported normal or corrected-to-normal vision, including those wearing contact lenses, and had no history of reading or neurological disorders. Participants who required glasses to read were excluded from the study. The study was approved by the Ethics Committee at Universidad Nebrija (Ref. UNNE-2023-0031). While fewer participants were tested than in Experiment 1, Experiment 2 included 66% more items, along with a two-line sentence display intended to reduce measurement error, both of which were expected to boost statistical power. Due to time and resource constraints, we recruited 24 participants. However, power simulations done after the study (see Appendix Fig. 10) suggest that power is still reasonably high due to the large number of sentences used.

#### Materials

The reading materials consisted of 200 sentences, arranged in 100 pairs. Each pair shared the same sentence structure at the beginning but differed in the lexical frequency of a single target word (high or low frequency) and sometimes in the context after the target word, based on the EsPal database (Duchon et al., 2013) for Spanish (see Table 5 for more information). The sentences were written specifically for the experiment and had a maximum length of 50 characters, including spaces and punctuation. The target word, always a noun, appeared in the middle of the sentence. An example item is given below:

**Table 5:** Psycholinguistic properties of the target words in Experiment 2 from the “EsPal” database (Duchon et al., 2013)

	High-frequency target words				Low-frequency target words			
	Mean	*SD*	Min	Max	Mean	*SD*	Min	Max
Lexical frequency (Zipf)	5.21	0.29	4.70	5.87	3.94	0.04	3.80	4
Word length	6.5	1.51	4	9	6.5	1.51	4	9



Target words ranged from four to nine letters in length and were carefully balanced across grammatical properties: half were feminine nouns and half were masculine nouns, and within each gender category, half were singular and half were plural. Thus, each frequency condition (high vs low) contained a balanced number of targets across gender and number.

The stimuli were divided into two lists: List A and List B. Each list contained 100 sentences: 50 with high-frequency target words and 50 with low-frequency target words. The other list contained the complementary items of each pair (i.e., the opposite frequency condition). Participants with odd-numbered IDs were assigned to List A, and those with even-numbered IDs to List B, ensuring that each participant saw only one version of each sentence pair. Within each list, sentence presentation order was randomised for each participant.

Sentences were displayed across two lines (no more than 25 characters per line) using a monospaced Courier New font, size 48, in black text on a white background. One third of the sentences were followed by a Yes/No comprehension question. Additionally, participants completed a short practice session before starting the experiment, consisting of five example sentences and three comprehension questions.

#### Apparatus

**Fig. 6:**
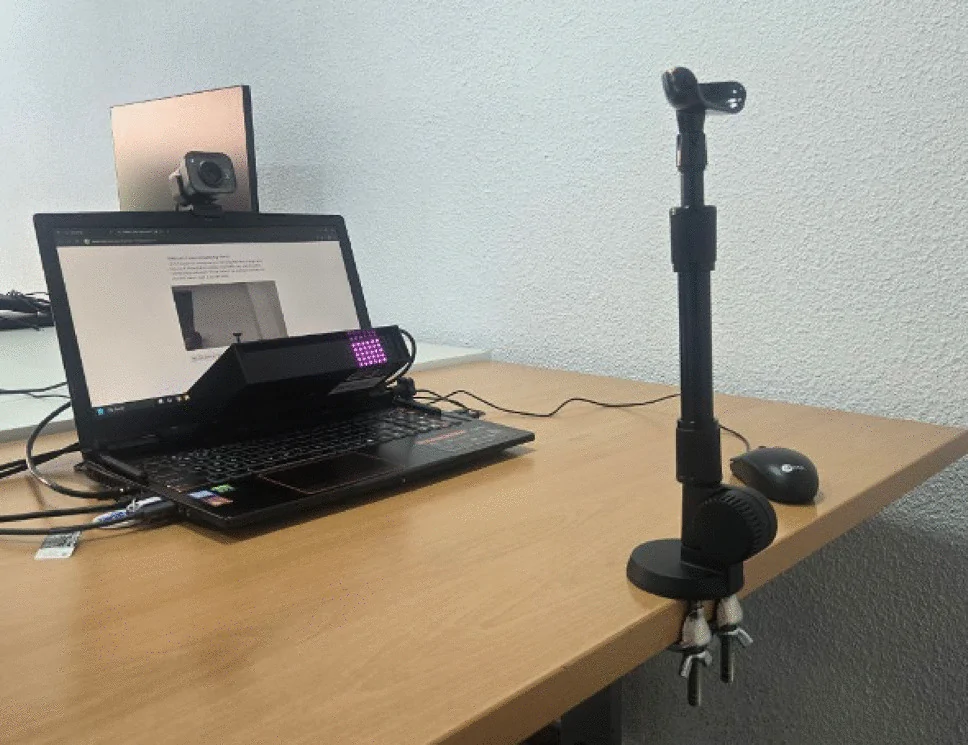
Apparatus set-up in Experiment 2

Eye movements were recorded simultaneously using an EyeLink Portable Duo eye tracker (SR Research, Ontario, Canada) at 1,000 Hz, and a Logitech StreamCam webcam at 60 Hz via the Labvanced platform (Kaduk et al., 2023) (see Fig. 6 for an illustration). The webcam achieved an effective sampling rate of 57.24 Hz on average (*SD* = 1.82 Hz; range, 53.24–59.72 Hz). Participants were seated in front of the laptop and used a chinrest to stabilise head movement. The eye-tracking set-up was run on a Windows 11 MSI Raider RGB laptop with a 24-inch LCD screen (resolution 1,920 × 1,080 pixels; refresh rate, 144 Hz; physical size, 35 × 20 cm). The eye-to-monitor distance was 63 cm. The webcam was positioned 58 cm from the eye and the EyeLink camera was positioned 53 cm from the eye. In this set-up, each pixel subtended 0.0162° horizontally and 0.0167° vertically. Each letter had a width of 55 pixels (0.89°).

The calibration procedure included two stages: first, a Labvanced webcam calibration with head movement tracking across various angles, followed by a nine-point calibration and validation procedure with the EyeLink system while the participant remained in the chinrest. The signals from the EyeLink and the webcam were synchronised offline using UNIX timestamps at the start of the experiment, and the EyeLink data were downsampled offline to match the effective webcam sampling rate, following the same procedure as Experiment 1.

#### Procedure

Participants gave written informed consent prior to participation. They were tested in a quiet room. After completing the dual calibration procedures (webcam and EyeLink), the experiment began. Participants first completed five practice trials, followed by the 100 experimental trials. There was no need to perform an additional drift-check during the trials, as the EyeLink calibration was monitored by the experimenter and remained stable in all cases; a seven-point drift correction grid was used to update the initial calibration model for the webcam every 25 trials. Additionally, the head position in relation to the webcam was monitored at all times, and the participant was asked to correct their posture if they moved too far.

Each trial began with a fixation cross (Shape ABC from Experiment 2 in Thaler et al., 2013, with a black outer circle, white cross, and red inner circle) at the position of the first letter of the sentence. After 250 ms, the sentence appeared. The sentences were presented in two left-aligned horizontal lines, each with a maximum length of 725 units (31.72 cm), such that each line was offset by 25 units (1.09 cm) from the left and right edges of the screen, respectively, occupying 90.63% of the total screen width in an 800 × 450 unit space. The first line was presented at the top of the screen, with a *y*-offset of 25 units (1.11 cm; 5.6% of screen height) from the top of the screen, and the second line was presented at a *y*-offset of 360 units (16.00 cm; ~80% of screen height) from the top of the screen. Participants read the sentence silently and clicked anywhere on the screen to continue to the next trial. In one third of the trials, a comprehension question followed the sentence, requiring a Yes/No mouse response. The entire session lasted between 20 and 30 min, including approximately 5–10 min for calibration and 10–15 min for the reading task.

#### Data analysis

The data were analysed in the same way as Experiment 1. The Engbert and Kliegl (2003) algorithm identified 20,265 fixations in the EyeLink data and 15,192 fixations in the webcam data. Samples that contained blinks (2.44%) or were otherwise outside the screen area (1.56%) were excluded from the data. Fixations shorter than 80 ms that were within one-character distance of a temporally adjacent fixation were merged into that adjacent fixation (0.21% [webcam], 1.65% [EyeLink]); any remaining fixations under 80 ms were discarded as outliers (4.30% [webcam], 16.2% [EyeLink]). Additionally, fixations longer than 1,000 ms were also discarded as outliers (1.23% [webcam], 0.31% [EyeLink]).

### Results and discussion

#### Comparison of raw sample positions

Participants’ average comprehension accuracy was 96.1% (*SD* = 19.35%; range, 83.3–100%), indicating that they read the sentences for understanding. The average total sentence reading time was 2,143 ms (*SD* = 899 ms). Person’s *r* correlations between the EyeLink and the webcam data are shown in Fig. 2. The sample correlations were *r* = 0.83 for the *x*-axis and *r* = 0.92 for the *y*-axis, indicating a slightly stronger association than in the Experiment 1 results, particularly for the *y*-dimension.

The Bland–Altman (1999) plots (see Fig. 3) showed very similar results to Experiment 1: there was little systematic bias in estimating the *x*-dimension, though the webcam again showed considerable variance in gaze positions. The *y*-dimension showed a weak negative trend, indicating that *y*-coordinates at the top of the screen tend to be slightly overestimated, whereas *y*-coordinates at the bottom of the screen tend to be slightly underestimated (see also Fig. 5b for a greater breakdown).

The average magnitude of the webcam error relative to the EyeLink was 0.10° (*SD* = 3.26°) for the *x*-dimension and 0.35° (*SD* = 2.32°) for the *y*-dimension (see Fig. 4b). This again shows little systematic bias, but a somewhat large variance in the error distribution. Notably, the *y*-dimension was estimated with much lower error and much less variance than in Experiment 1. This suggests that the set-up in Experiment 2 (using a laptop and only two lines of text) led to more accurate tracking of *y*-positions.

The distribution of errors across screen regions showed similar trends to Experiment 1 (see Fig. 5b). Horizontal error was smallest at the centre of the screen, with the webcam overestimating gaze positions to the left and underestimating them to the right of the centre. The positive and negative horizontal errors again largely cancelled each other out. The vertical errors were more mixed, but again showed a slight overestimation bias, particularly at the top of the screen. The bottom of the screen showed the least amount of error, though gaze positions were slightly underestimated there. Vertical error was largest at the centre of the screen; however, because no text was presented there, this likely reduced its impact on the analysis of reading behaviour.

#### Analysis of target word lexical frequency effect

Table 6 shows the means and standard deviations for the dependent measures on target words in Experiment 2, while Table 7 shows the corresponding model estimates.

**Table 6:** Mean first fixation duration (FFD), single fixation duration (SFD), gaze duration (GD), total viewing time (TVT), and first-pass skipping probability on target words in Experiment 2 by eye tracker and target word frequency condition

		FFD		SFD		GD		TVT		Skipping probability	
Tracker	Frequency	Mean	*SD*	Mean	*SD*	Mean	*SD*	Mean	*SD*	Mean	*SD*
Webcam	Low	236	119	239	120	249	125	293	173	0.22	0.42
Webcam	High	219	104	222	105	236	119	277	192	0.25	0.44
EyeLink	Low	160	67	163	69	183	87	210	114	0.14	0.35
EyeLink	High	153	59	155	59	175	84	203	122	0.17	0.38

*FFD* first fixation duration; *SFD* single fixation duration; *GD* gaze duration; *TVT* total viewing time; *Skipping* First-pass skipping probability; *SD* standard deviation. All means and standard deviations are reported in milliseconds (ms)

**Table 7:** Bayesian linear mixed-effects models showing the target word lexical frequency effect in Experiment 2. The results show coefficient estimates and 95% credible intervals (in square brackets)

	FFD	SFD	GD	TVT	Skipping probability
Intercept	**5.163**	**5.188**	**5.243**	**5.353**	**−1.707**
	[**5.105, 5.221**]	[**5.118, 5.257**]	[**5.164, 5.321**]	[**5.254, 5.451**]	[**−1.990, −1.433**]
Frequency	**0.060**	**0.057**	**0.060**	**0.070**	−0.190
	[**0.021, 0.099**]	[**0.015, 0.099**]	[**0.013, 0.108**]	[**0.014, 0.126**]	[−0.665, 0.305]
Tracker	**0.359**	**0.362**	**0.317**	**0.314**	**0.532**
	[**0.298, 0.422**]	[**0.298, 0.427**]	[**0.264, 0.371**]	[**0.250, 0.379**]	[**0.042, 1.006**]
Frequency × Tracker	0.033	0.028	0.014	0.032	−0.074
	[−0.018, 0.084]	[−0.024, 0.080]	[−0.039, 0.065]	[−0.028, 0.092]	[−0.448, 0.309]

Effects for which the 95% CrI excludes 0 are highlighted in bold. *FFD* first fixation duration; *SFD* single fixation duration; *GD* gaze duration; *TVT* total viewing time

As in Experiment 1, we observed a frequency effect in the expected direction across both devices: low-frequency words elicited longer fixations than high-frequency words (FFD, b=0.06, 95% CrI $\left[0.02,\hspace{0.25em}0.10\right]$, $P\left(b>0\right)=1.00$, $E{R}_{b>0}=550.72$, $B{F}_{10}=1.51$; SFD, b=0.06, 95% CrI $\left[0.01,\hspace{0.25em}0.10\right]$, $P\left(b>0\right)=1.00$, $E{R}_{b>0}=218.18$, $B{F}_{10}=0.70$; GD, b=0.06, 95% CrI $\left[0.01,\hspace{0.25em}0.11\right]$, $P\left(b>0\right)=0.99$, $E{R}_{b>0}=155.86$, $B{F}_{10}=0.50$; TVT, b=0.07, 95% CrI $\left[0.01,\hspace{0.25em}0.13\right]$, $P\left(b>0\right)=0.99$, $E{R}_{b>0}=124.98$, $B{F}_{10}=0.60$). Skipping probability was lower for low-frequency than high-frequency words, though the effect was not credible (b=-0.19, 95% CrI $-\left[0.66,\hspace{0.25em}0.30\right]$, $P\left(b<0\right)=0.79$, $E{R}_{b<0}=3.67$, $B{F}_{10}=0.33$). Fixation times were again longer and skipping probabilities were higher with the webcam than with the EyeLink (FFD, b=0.36, 95% CrI $\left[0.30,\hspace{0.25em}0.42\right]$, $P\left(b>0\right)=1.00$, $E{R}_{b>0}>\mathrm{1,000}$, $B{F}_{10}>\mathrm{1,000}$; SFD, b=0.36, 95% CrI $\left[0.30,\hspace{0.25em}0.43\right]$, $P\left(b>0\right)=1.00$, $E{R}_{b>0}>\mathrm{1,000}$, $B{F}_{10}>\mathrm{1,000}$; GD, b=0.32, 95% CrI $\left[0.26,\hspace{0.25em}0.37\right]$, $P\left(b>0\right)=1.00$, $E{R}_{b>0}>\mathrm{1,000}$, $B{F}_{10}>\mathrm{1,000}$; TVT, b=0.31, 95% CrI $\left[0.25,\hspace{0.25em}0.38\right]$, $P\left(b>0\right)=1.00$, $E{R}_{b>0}>\mathrm{1,000}$, $B{F}_{10}>\mathrm{1,000}$; skipping, b=0.53, 95% CrI $\left[0.04,\hspace{0.25em}1.01\right]$, $P\left(b>0\right)=0.98$, $E{R}_{b>0}>59.84$, $B{F}_{10}>2.26$). Again, we did not observe a credible frequency × tracker interaction (FFD, b=0.03, 95% CrI $\left[-0.02,\hspace{0.25em}0.08\right]$, $P\left(b>0\right)=0.90$, $E{R}_{b>0}=8.96$, $B{F}_{10}=0.06$; SFD, b=0.03, 95% CrI $\left[-0.02,\hspace{0.25em}0.08\right]$, $P\left(b>0\right)=0.86$, $E{R}_{b>0}=6.05$, $B{F}_{10}=0.05$; GD, b=0.01, 95% CrI $\left[-0.04,\hspace{0.25em}0.07\right]$, $P\left(b>0\right)=0.69$, $E{R}_{b>0}=2.27$, $B{F}_{10}=0.03$; TVT, b=0.03, 95% CrI $\left[-0.03,\hspace{0.25em}0.09\right]$, $P\left(b>0\right)=0.86$, $E{R}_{b>0}=6.20$, $B{F}_{10}=0.05$; skipping, b=-0.07, 95% CrI $\left[-0.45,\hspace{0.25em}0.31\right]$, $P\left(b<0\right)=0.65$, $E{R}_{b<0}=1.84$, $B{F}_{10}=0.21$).

Similar to Experiment 1, we summarised the low- versus high-frequency contrast separately within each device using estimated marginal means. Interestingly, in Experiment 2, the webcam data contained stronger evidence in favour of the frequency effect than the EyeLink data, with the 95% CI for the frequency effect excluding 0 for both devices in FFD (webcam, *M* = 0.08, 95% CrI [0.03, 0.13]; EyeLink, *M* = 0.04, 95% CrI [0.00, 0.09]) and GD (webcam, *M* = 0.07, 95% CrI [0.01, 0.12]; EyeLink, *M* = 0.05, 95% CrI [−0.00, 0.11]), although the exclusion was very narrow for the EyeLink data), but only for the webcam data in SFD (webcam, *M* = 0.07, 95% CrI [0.02, 0.12]; EyeLink, *M* = 0.04, 95% CrI [−0.00, 0.09]) and TVT (webcam, *M* = 0.09, 95% CrI [0.02, 0.15]; EyeLink, *M* = 0.05, 95% CrI [−0.01, 0.12]). The marginal effect of skipping probability was not credible for either the EyeLink or the webcam data, though the difference was in the expected direction (webcam, *M* = −0.22, 95% CrI [−0.73, 0.29]; EyeLink, *M* = −0.15, 95% CrI [−0.67, 0.38]). These somewhat unexpected results show that the webcam data, although noisier, do not necessarily always underestimate the size of an effect. In any case, our results highlight that a large number of observations are needed to reliably detect even strong effects such as the word frequency effect at a sampling rate of 30 Hz (as suggested by Angele et al., 2025).

#### Analysis of corpus lexical frequency

For Experiment 2, we also fit models predicting fixation times on all the words in every sentence (except for sentence-initial and sentence-final words), with Zipf frequency as a continuous predictor and tracker as a discrete factor with two levels, EyeLink and webcam, as was done for the Experiment 1 data. Table 8 summarises the Zipf frequency effect, the effect of the tracker used, and their interaction over all eligible words. We again observed a frequency effect, with higher-frequency words being associated with shorter fixations and higher skipping probability (FFD, b=-0.03, 95% CrI $\left[-0.04,\hspace{0.25em}-0.02\right]$, $P\left(b<0\right)=1.00$, $E{R}_{b<0}>\mathrm{1,000}$, $B{F}_{10}>\mathrm{1,000}$; SFD, b=-0.04, 95% CrI $\left[-0.05,\hspace{0.25em}-0.02\right]$, $P\left(b<0\right)=1.00$, $E{R}_{b<0}>\mathrm{1,000}$, $B{F}_{10}>\mathrm{1,000}$; GD, b=-0.06, 95% CrI $\left[-0.08,\hspace{0.25em}-0.05\right]$, $P\left(b<0\right)=1.00$, $E{R}_{b<0}>\mathrm{1,000}$, $B{F}_{10}>\mathrm{1,000}$; TVT, b=-0.09, 95% CrI $\left[-0.10,\hspace{0.25em}-0.07\right]$, $P\left(b<0\right)=1.00$, $E{R}_{b<0}>\mathrm{1,000}$, $B{F}_{10}>\mathrm{1,000}$; skipping, b=0.92, 95% CrI $\left[0.83,\hspace{0.25em}1.01\right]$, $P\left(b>0\right)=1.00$, $E{R}_{b>0}>\mathrm{1,000}$, $B{F}_{10}>\mathrm{1,000}$). Just as we had observed in all the previous analyses, the webcam data resulted in longer detected fixations overall (FFD, b=0.44, 95% CrI $\left[0.39,\hspace{0.25em}0.50\right]$, $P\left(b>0\right)=1.00$, $E{R}_{b>0}>\mathrm{1,000}$, $B{F}_{10}>\mathrm{1,000}$; SFD, b=0.44, 95% CrI $\left[0.38,\hspace{0.25em}0.49\right]$, $P\left(b>0\right)=1.00$, $E{R}_{b>0}>\mathrm{1,000}$, $B{F}_{10}>\mathrm{1,000}$; GD, b=0.39, 95% CrI $\left[0.35,\hspace{0.25em}0.44\right]$, $P\left(b>0\right)=1.00$, $E{R}_{b>0}>\mathrm{1,000}$, $B{F}_{10}>\mathrm{1,000}$; TVT, b=0.36, 95% CrI $\left[0.32,\hspace{0.25em}0.41\right]$, $P\left(b>0\right)=1.00$, $E{R}_{b>0}>\mathrm{1,000}$, $B{F}_{10}>\mathrm{1,000}$). Skipping probability was also higher in the webcam data, though the effect was not credible (b=0.22, 95% CrI $\left[-0.02,\hspace{0.25em}0.46\right]$, $P\left(b>0\right)=0.96$, $E{R}_{b>0}>24.93$, $B{F}_{10}=0.59$).

**Table 8:** Bayesian linear mixed-effects models showing the corpus lexical frequency effect on all words (except for the first and last in each sentence) in Experiment 2. The results show coefficient estimates and 95% credible intervals (in square brackets)

	FFD	SFD	GD	TVT	Skipping probability
Intercept	**5.166**	**5.183**	**5.244**	**5.344**	**−0.416**
	[**5.113, 5.219**]	[**5.124, 5.241**]	[**5.178, 5.311**]	[**5.269, 5.424**]	[**−0.609, −0.225**]
Zipf frequency	**−0.029**	**−0.037**	**−0.061**	**−0.085**	**0.919**
	[**−0.041, −0.018**]	[**−0.051, −0.023**]	[**−0.077, −0.046**]	[**−0.103, −0.067**]	[**0.828, 1.009**]
Tracker	**0.444**	**0.436**	**0.395**	**0.364**	0.217
	[**0.390, 0.503**]	[**0.383, 0.489**]	[**0.348, 0.442**]	[**0.316, 0.411**]	[−0.025, 0.455]
Zipf Frequency × Tracker	0.010	**0.014**	**0.032**	**0.026**	**−0.273**
	[−0.002, 0.022]	[**0.001, 0.026**]	[**0.021, 0.044**]	[**0.014, 0.037**]	[**−0.328, −0.220**]

Effects for which the 95% CrI excludes 0 are highlighted in bold. *FFD* first fixation duration; *SFD* single fixation duration; *GD* gaze duration; *TVT* total viewing time

 There was no credible evidence for an interaction between Zipf frequency and tracker in FFD (b=0.0096, 95% CrI $\left[-0.0025,\hspace{0.25em}0.02\right]$, $P\left(b>0\right)=0.94$, $E{R}_{b>0}=15.21$, $B{F}_{10}=0.02$), but there was a credible interaction in SFD (b=0.01, 95% CrI $\left[0.0014,\hspace{0.25em}0.03\right]$, $P\left(b>0\right)=0.99$, $E{R}_{b>0}=66.51$, $B{F}_{10}=0.07$), GD (b=0.03, 95% CrI $\left[0.02,\hspace{0.25em}0.04\right]$, $P\left(b>0\right)=1.00$, $E{R}_{b>0}>\mathrm{1,000}$, $B{F}_{10}>\mathrm{1,000}$), TVT (b=0.03, 95% CrI $\left[0.01,\hspace{0.25em}0.04\right]$, $P\left(b>0\right)=1.00$, $E{R}_{b>0}>\mathrm{1,000}$, $B{F}_{10}=11.10$), and skipping (b=-0.27, 95% CrI $\left[-0.33,-0.22\right]$, $P\left(b<0\right)=1.00$, $E{R}_{b<0}>\mathrm{1,000}$, $B{F}_{10}>\mathrm{1,000}$). Unlike the target word analysis, the frequency effect was stronger in the EyeLink data than the webcam data. Finally, we again report *emtrends* estimates and 95% CIs for EyeLink and webcam data separately. Just like Experiment 1, the frequency effect was credible for both the EyeLink and the webcam data in all measures (FFD: EyeLink, *M* = −0.03, 95% CrI [−0.05, −0.02]; webcam, *M* = −0.02, 95% CrI [−0.04, −0.01]; SFD: EyeLink, *M* = −0.04, 95% CrI [−0.06, −0.03]; webcam, *M* = −0.03, 95% CrI [−0.05, −0.01]; GD: EyeLink, *M* = −0.08, 95% CrI [−0.09, −0.06]; webcam, *M* = −0.05, 95% CrI [−0.06, −0.03]; TVT: EyeLink, *M* = −0.10, 95% CrI [−0.12, −0.08]; webcam, *M* = −0.07, 95% CrI [−0.09, −0.05]; skipping: EyeLink, *M* = 1.06 95% CrI [0.96, 1.15]; webcam, *M* = 0.78 95% CrI [0.69, 0.87]). 

#### Summary and conclusions

To summarise, while Experiment 2 replicated many of the key findings from Experiment 1, the data also showed stronger effects in some cases. First, the webcam and EyeLink data exhibited similar, but slightly higher, positive correlations. This again suggests that gaze coordinates recorded by the webcam are broadly similar to those recorded by the EyeLink. Nevertheless, similar to Experiment 1, the webcam data still showed considerable variance in the error distributions, although there was little systematic bias and the *y*-dimension was estimated more accurately than in Experiment 1. Therefore, these results suggest that the set-up employed in Experiment 2 (where the stimuli were presented on a laptop screen and were displayed over two lines of text) results in more accurate webcam tracking.

With regard to the reading measures, the data were again stronger, in the sense that the target word lexical frequency effect was now also credibly detected in the webcam data. Surprisingly, the webcam data showed numerically larger effects than the EyeLink data, though the means were also estimated with much more variance. In the corpus frequency analysis, both the webcam and EyeLink showed reliable frequency effects, replicating Experiment 1. Therefore, Experiment 2 showed similar though stronger frequency effects in the webcam data. It is also worth noting that the frequency effect on word skipping was credible only in the corpus and not in the target word analysis. This suggests that skipping effects may be harder to estimate at low sampling rates than fixation duration effects.

Taken together, Experiments 1 and 2 show that frequency effects can *in principle* be detected with webcams under lab conditions. However, it is not clear whether similar results can also be obtained in the “real world”, where the experimenter has less control over the testing protocol and participants have webcams with a lower sampling resolution (typically 30 Hz vs the 60 Hz used in the lab studies). To help answer this question, we repeated Experiment 1 online, with participants completing the study at home.^5^

## Experiment 3

### Method

Sixty-six participants (32 female, 33 male, 1 preferred not to say) were recruited from Prolific.com for payment of £10/h. Participants reported having English as their native language, normal or corrected-to-normal vision (with contact lenses), and no prior history of reading disorders. Participants’ average age was 32.04 years (*SD* = 7.86; range, 18–50). Participants’ self-reported ethnicity was as follows: White (65.15%), Black (21.21%), Asian (10.61%), and mixed (3.03%). A larger number of participants was recruited than in Experiment 1 because we expected greater variability in the data due to the less standardised testing conditions. Therefore, we increased the sample size by roughly a third.

Participants completed the same study as Experiment 1 at home. The first 18 participants were run as a pilot. They completed only the lexical frequency corpus task (the single-line corpus was used to pilot another task, which was not included in the final study). Therefore, the data for the single-sentence corpus were based only on 48 participants, while all 66 participants completed the lexical frequency corpus. The average effective sampling rate in the study was 25.66 Hz (*SD* = 6.70 Hz; range, 9.66–35.46 Hz).^6^ The rest of the method was identical to Experiment 1.

The data analysis was identical to the previous experiments. However, because participants completed the study at home, no EyeLink data are available. Therefore, the statistical models contained only Frequency as the predictor. The only difference from previous experiments is that the saccade detection threshold λ of Engbert and Kliegl’s (2003) algorithm was reduced from 6 to 3 to deal with the lower data sampling rate (30 Hz). Using a threshold of λ = 6 led to fewer and excessively long fixations; reducing it to λ = 3 improved the sensitivity of the algorithm and led to fixation durations that were more similar to Experiments 1 and 2. The algorithm identified 35,610 fixations in the webcam data (29,626 in the Frequency and 5,984 in the Single-line corpus).

Samples that contained blinks (4.91%) or were otherwise outside the screen area (1.36%) were removed from analysis. Fixations shorter than 80 ms that were within one-character distance of a temporally adjacent fixation were merged into that adjacent fixation (0.59%); any remaining fixations under 80 ms were discarded as outliers (6.94%). Additionally, fixations longer than 1,000 ms were also discarded as outliers (5.78%).

#### Data analysis

The model specifications and data analysis were the same as in Experiment 1, except that Tracker was not included as a predictor since all data were collected remotely using the participants’ webcam.

### Results and discussion

Participants’ comprehension accuracy was 95.9% (*SD* = 19.6%; range, 85–100%), showing that they read for understanding. The average total sentence reading time was 3,864 ms (*SD* = 2,801 ms).

#### Analysis of target word lexical frequency effect

Table 9 shows the mean and standard deviation of first fixation duration (FFD), single fixation duration (SFD), gaze duration (GD), total viewing time (TVT), and skipping probability on target words in Experiment 3 by word frequency condition, while Table 10 shows the model estimates.

**Table 9:** Mean first fixation duration (FFD), single fixation duration (SFD), gaze duration (GD), total viewing time (TVT), and first-pass skipping probability on target words in Experiment 3 by target word frequency condition

	FFD		SFD		GD		TVT		Skipping probability	
Frequency	Mean	*SD*	Mean	*SD*	Mean	*SD*	Mean	*SD*	Mean	*SD*
Low	381	226	385	227	436	279	521	372	0.53	0.50
High	363	226	359	226	401	271	493	386	0.50	0.50

*FFD* first fixation duration; *SFD* single fixation duration; *GD* gaze duration; *TVT* total viewing time; *SD* standard deviation. All means and standard deviations are reported in milliseconds (ms)

**Table 10:** Bayesian linear mixed-effects models showing the target word lexical frequency effect in Experiment 1. The results show coefficient estimates and 95% credible intervals (in square brackets)

	FFD	SFD	GD	TVT	Skipping probability
Intercept	**5.761**	**5.754**	**5.853**	**5.988**	0.104
	[**5.681, 5.840**]	[**5.677, 5.834**]	[**5.775, 5.933**]	[**5.912, 6.065**]	[−0.105, 0.321]
Frequency	**0.075**	**0.090**	**0.112**	**0.095**	0.105
	[**0.018, 0.130**]	[**0.028, 0.153**]	[**0.049, 0.173**]	[**0.028, 0.160**]	[−0.037, 0.249]

Effects for which the 95% CrI excludes 0 are highlighted in bold. *FFD* first fixation duration; *SFD* single fixation duration; *GD* gaze duration; *TVT* total viewing time

In Experiment 3, low-frequency target words elicited longer fixation times than high-frequency target words in FFD (b=0.07, 95% CrI $\left[0.02,\hspace{0.25em}0.13\right]$, $P\left(b>0\right)=0.99$, $E{R}_{b>0}=180.82$, $B{F}_{10}=0.76$), SFD (b=0.09, 95% CrI $\left[0.03,\hspace{0.25em}0.15\right]$, $P\left(b>0\right)=1.00$, $E{R}_{b>0}=371.09$, $B{F}_{10}=1.81$), GD (b=0.11, 95% CrI $\left[0.05,\hspace{0.25em}0.17\right]$, $P\left(b>0\right)=1.00$, $E{R}_{b>0}>\mathrm{1,000}$, $B{F}_{10}=6.17$), and TVT (b=0.09, 95% CrI $\left[0.03,\hspace{0.25em}0.16\right]$, $P\left(b>0\right)=1.00$, $E{R}_{b>0}=312.73$, $B{F}_{10}=1.38$). For skipping probability, the CrI included 0, although the effect was numerically in the expected direction (b=0.11, 95% CrI $\left[-0.04,\hspace{0.25em}0.25\right]$, $P\left(b>0\right)=0.93$, $E{R}_{b>0}=12.72$, $B{F}_{10}=0.20$). Overall, this demonstrates that, despite the much lower control we had over devices and experimental set-up in the online study, reliable word frequency effects could still be found in fixation duration measures.

### Analysis of corpus lexical frequency

As in Experiment 1, we also fitted Bayesian linear mixed models including all words in every sentence (both the multi-line sentences with the frequency manipulation and the single-line sentences) into the analysis. This increased the number of observations substantially compared with the target word analysis.

Table 11 shows the results of these “corpus-analysis style” Bayesian linear mixed models with Zipf frequency as a continuous predictor for the four fixation time measures on all words in the sentences (except for the first and last word in each sentence) in Experiment 3.

**Table 11:** Bayesian linear mixed-effects models showing the corpus lexical frequency effect on all words (except for the first and last in each sentence) in Experiment 3. The results show coefficient estimates and 95% credible intervals (in square brackets)

	FFD	SFD	GD	TVT	Skipping probability
Intercept	**5.646**	**5.656**	**5.704**	**5.820**	**1.062**
	[**5.582, 5.714**]	[**5.590, 5.723**]	[**5.636, 5.771**]	[**5.762, 5.878**]	[**0.916, 1.205**]
Zipf frequency	**−0.038**	**−0.039**	**−0.060**	**−0.077**	**0.293**
	[**−0.050, −0.027**]	[**−0.051, −0.027**]	[**−0.072, −0.048**	[**−0.090, −0.063**]	[**0.233, 0.352**]

Effects for which the 95% CrI excludes 0 are highlighted in bold. *FFD* first fixation duration; *SFD* single fixation duration; *GD* gaze duration; *TVT* total viewing time

In Experiment 3, we observed a consistent effect of Zipf frequency on all measures (FFD, b=-0.04, 95% CrI $\left[-0.05,\hspace{0.25em}-0.03\right]$, $P\left(b<0\right)=1.00$, $E{R}_{b<0}>\mathrm{1,000}$, $B{F}_{10}>\mathrm{1,000}$; SFD, b=-0.04, 95% CrI $\left[-0.05,\hspace{0.25em}-0.03\right]$, $P\left(b<0\right)=1.00$, $E{R}_{b<0}>\mathrm{1,000}$, $B{F}_{10}>\mathrm{1,000}$; GD, b=-0.06, 95% CrI $\left[-0.07,\hspace{0.25em}-0.05\right]$, $P\left(b<0\right)=1.00$, $E{R}_{b<0}>\mathrm{1,000}$, $B{F}_{10}>\mathrm{1,000}$; TVT, b=-0.08, 95% CrI $\left[-0.09,\hspace{0.25em}-0.06\right]$, $P\left(b<0\right)=1.00$, $E{R}_{b<0}>\mathrm{1,000}$, $B{F}_{10}>\mathrm{1,000}$; skipping, b=0.29, 95% CrI $\left[0.23,\hspace{0.25em}0.35\right]$, $P\left(b>0\right)=1.00$, $E{R}_{b>0}>\mathrm{1,000}$, $B{F}_{10}>\mathrm{1,000}$). This again demonstrates that, when there is a sufficiently high number of observations, the frequency effect can be detected using online webcam eye tracking, even though we did not have direct control over the experimental set-up and the sampling rate of participants’ cameras was generally lower. It is also noteworthy that the frequency effect in skipping probability was now credible in the corpus analysis.

#### Summary and conclusions

In summary, the eye-tracking data obtained online without any direct experimenter control of the testing environment were clearly noisier than the laboratory-based data. Despite this, we found reliable word frequency effects, both in terms of the categorical effect of the target word manipulation and in terms of the continuous effect of Zipf frequency on all words. This shows that effects of cognitive processes on eye movements can be measured even in fully online eye-tracking studies if the sample size is large enough. Still, the target word frequency effect in skipping probability was not credible, suggesting that experimental effects may be harder to capture in this measure. Nevertheless, the fact that a skipping effect was observed in the corpus analysis with all words suggests that this limitation can be overcome with a larger number of observations.

**Fig. 7:**
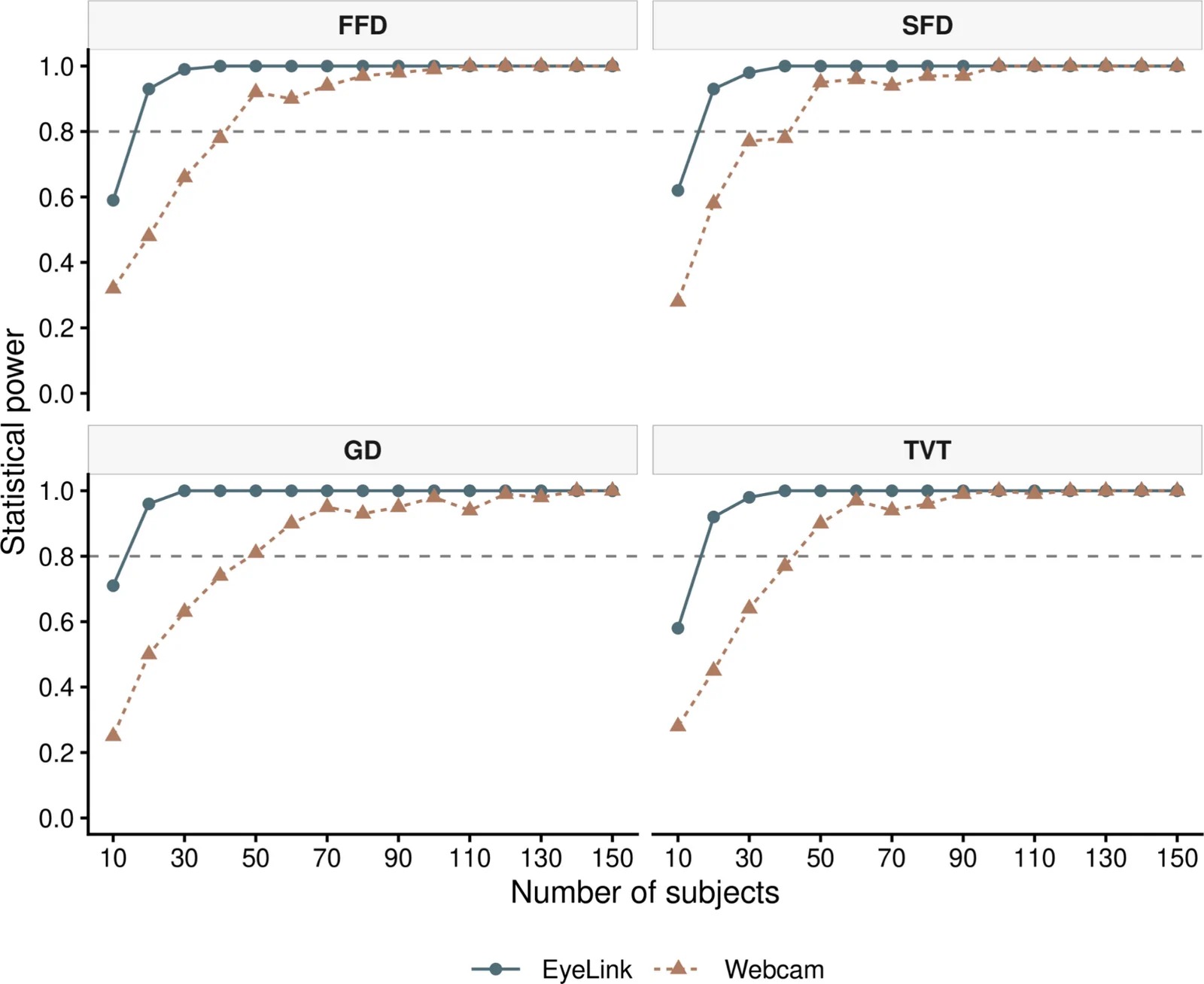
Statistical simulations showing the empirical power to detect the target word lexical frequency effect in the webcam data from Experiment 3 and the EyeLink data from Experiment 1. The 80% power level is indicated by a horizontal grey line. Simulations were conducted with the *simr* package (Green & MacLeod, 2016) based on 60 experimental items (same as in Experiments 1 and 3). Power was calculated with 100 simulations per cell and included 10% random data loss. The models included random intercepts for subjects and items, and random slopes for target word frequency.

To aid sample size planning for future studies, we estimated statistical power for the lexical frequency effect using simulations with the *simr* package (Green & MacLeod, 2016). Figure 7 shows the statistical power needed to detect the target word lexical frequency effect based on the webcam results from Experiment 3. Simulations based on the EyeLink results from Experiment 1 are also added for comparison. The results show that 60 participants and 60 items are sufficient to achieve 80% statistical power for detecting the lexical frequency effect in fixation duration measures for the webcam data (see Figure 10 in the Appendix for a full breakdown). However, researchers should perform their own calculations for other effects. The effect in skipping probability was more difficult to capture, but the simulations suggested that at least 130 subjects and 130 items are needed to reach 80% power (see the Appendix).

## General discussion

We performed three experiments to test whether it is feasible to study eye movements during sentence reading using webcam-based eye tracking as implemented in the Labvanced experimental platform. We were particularly interested in whether we could observe the word frequency effect, as this requires assigning fixations accurately to word-based regions of interest. In Experiments 1 and 2, we compared the performance of webcam eye tracking as implemented in Labvanced with the SR Research EyeLink under controlled laboratory conditions. In Experiment 3, we recruited participants on an online platform to participate in an eye-tracking experiment using their own devices outside of the laboratory. In each of these experiments, we were able to find evidence for the word frequency effect in the webcam data, although the strength of the evidence varied by experimental set-up and design. In the lab-based Experiments 1 and 2 that co-registered webcam and EyeLink data, we found that, as expected, the webcam data were noisier than those recorded by the high-precision EyeLink eye tracker. Despite this, there was a surprisingly high correlation between the data sources in terms of the estimated gaze position and, importantly, there was little systematic bias observed in the webcam data, especially in the *x* dimension, which is critically important for determining which word in a sentence a reader is fixating on. The relative absence of large directional biases means that we can reduce the amount of noise by averaging over multiple observations, compensating for the lower quality of the webcam data by increasing the sample size (similar to the results reported by Angele et al., 2025, who found that the increased noise associated with tracking at lower sampling rates can be compensated by increasing the number of observations). Therefore, our results coincide with those of Kaduk et al. (2023), who also found high correlations between the gaze position estimates of the webcam and EyeLink in a free viewing task.

Of course, the focus of the current study was whether we could obtain useful information about fixation times on words in a reading task. The lower precision of the webcam data made it necessary to adapt the experimental design relative to how lab-based studies with high-precision eye trackers are normally conducted. Instead of being able to present a long sentence using a medium to small font size in a single, vertically centred line, we used a much larger font size, either severely limiting the sentence length (such as in the single-line sentences presented in Experiments 1 and 3) or resulting in the sentences being split across multiple lines. We know that line breaks and the resulting return sweeps affect reading behaviour (Parker et al., 2019), so this represents a limitation that cannot be easily addressed. On the other hand, a great deal of reading nowadays takes place on mobile devices with a similarly limited number of characters or words per line, although typically with a much smaller font size than the one used here. Regarding the impact on reading behaviour specifically, we designed our materials to minimise the influence of return sweeps on our critical measure: the target word was never the first or last word on its line, so the frequency effect reported here is unlikely to be confounded by line-boundary artifacts. Other words in the sentence may still show the return-sweep-related effects described by Parker et al. (2019), potentially altering participants’ overall reading behaviour relative to single-line text. Comprehension accuracy was high overall across all experiments (> 95%), suggesting participants could extract sentence-level meaning despite the multi-line format, although our comprehension questions assessed only immediate understanding and cannot speak to longer-term retention. Additionally, the assignment of fixations to lines in multi-line text is not trivial and can be the source of additional errors and/or require the application of correction algorithms (Carr et al., 2022; Mercier et al., 2024). In the present study, we did not apply any line correction algorithms and still obtained evidence of the frequency effect.

In Experiment 2, we attempted to strike a balance between sentence length, font size, and the ease of assigning fixations to lines by splitting each sentence into two lines and presenting them at the top and towards the bottom of the screen, respectively. This may be a satisfactory compromise when using webcam eye tracking for medium-length sentences, although surely our design can be further refined in the future.

A strong correlation between webcam and EyeLink positional data is necessary, but not sufficient, for generating similar fixation-time measures. Because fixations are assigned to words based on bounding boxes around them, the key factor is whether gaze samples fall within the correct word region. In this context, even small positional errors can shift a fixation into an adjacent word and introduce substantial noise, whereas larger errors may have little impact if they remain within the correct word boundaries. Despite this concern, we found that the EyeLink and the webcam data showed frequency effects in the same direction for fixation duration measures, even though these were not always statistically reliable in the noisier webcam data (e.g., Experiment 1). As described above, collecting more data can help with this issue: in the corpus-style analyses which included observations from most of the words in each sentence, the evidence for the frequency effect was much clearer in the webcam data than it was in the analyses of the experimental target word frequency manipulation. The latter involved a stronger experimental control of the sentence context and target word properties but resulted in fewer observations, as only one word per sentence could be analysed. This is not to say that experimental designs cannot be done using online eye tracking, but researchers should plan to collect data from many participants and, ideally, use many items to reach a high number of observations per condition.

**Fig. 8:**
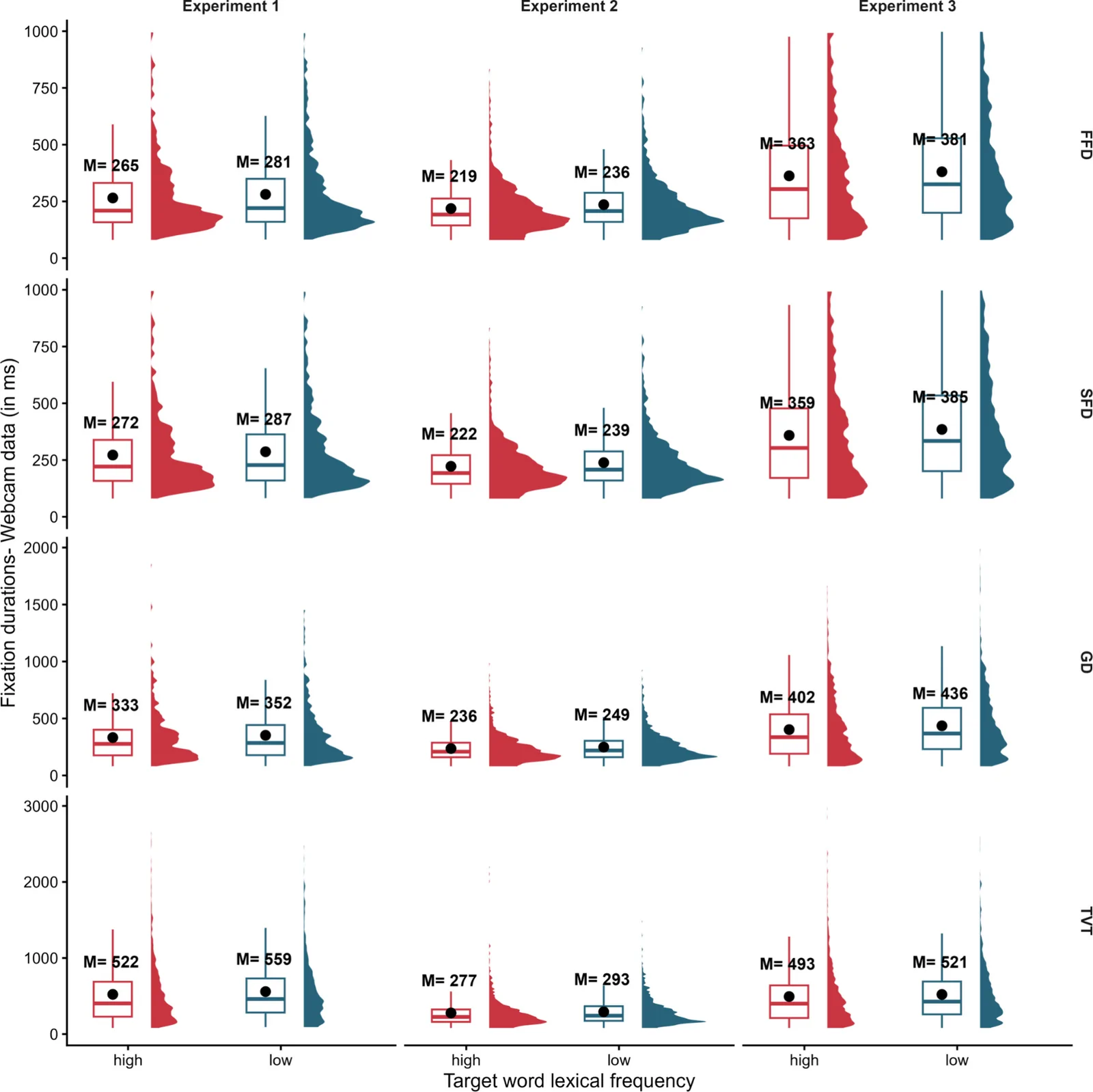
Fixation durations in the webcam data, showing the target word lexical frequency effect in Experiments 1–3. The graph shows the boxplots on the left and the density distributions on the right. Black dots show the mean, whereas the boxplots show the median

**Fig. 10:**
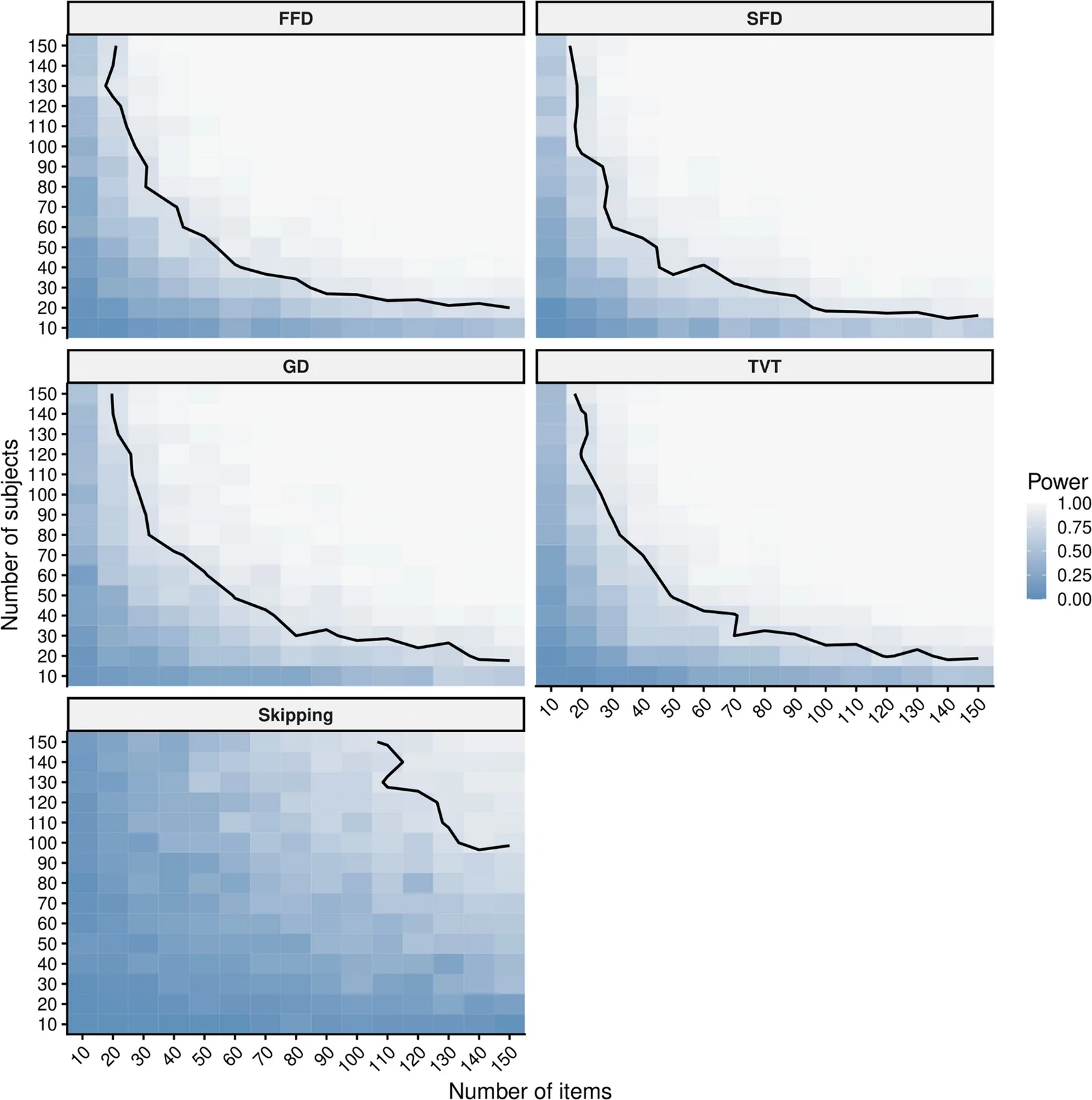
Power spectrum plots for the webcam data based on the results from Experiment 3. Power levels were computed for different subject and item numbers (ranging between 10 and 150, in intervals of 10). The 80% power level is indicated by solid black contour lines. Simulations were conducted with the *simr* package (Green & MacLeod, 2016). Power was calculated with 100 simulations per cell and included 10% random data loss. The models included random intercepts for subjects and items, and random slopes for target word frequency

Overall, we observed clear evidence of the word frequency effect in all experiments. The target word frequency effect was numerically similar in all experiments in the webcam data for fixation duration measures (see Fig. 8). While the target word frequency effect was not credible in Experiment 1, the corpus-level analysis nevertheless showed evidence for a corpus frequency effect. However, it is important to note that the frequency effect was captured less well in skipping probability. Indeed, skipping rates were substantially higher in the webcam data than the EyeLink. This likely reflects the tendency of the webcam data to merge nearby fixations, meaning that smaller saccades can sometimes be missed. While frequency effects in skipping were present in the corpus analyses, webcams may be less well suited to capture this measure.

Our fixation duration measures generally replicated the findings by Angele et al. (2025), who, based on downsampling high-precision EyeLink data, predicted that it should be possible to detect the word frequency effects even at sampling rates as low as 60 or 30 Hz. By finding the frequency effect in the webcam data, we confirmed their prediction that this would also be the case with non-downsampled data that were recorded at lower sampling rates.

An important finding from Experiment 2 is that the noisier webcam data do not always necessarily underestimate the effect size: while noise is likely to result in weaker evidence for an effect, sometimes the noise in the data can result in a larger effect size estimate. A larger sample size should also help with obtaining a more accurate effect size estimate by reducing variance around the means.

Experiment 3 demonstrated that running an eye tracking experiment completely online with 30 Hz webcams, with all the variability this implies in terms of environment, device, participant compliance with experimenter instructions, etc., does result in noisier data, but this did not prevent us from finding evidence for the word frequency effect in fixation duration measures. Indeed, downsampling the data from Experiments 1 and 2 to 30 Hz resulted in comparable target word frequency results (see Tables S1 to S4 in the Supplementary Materials). This suggests that the low sampling rate of most webcams is not an insurmountable barrier to collecting usable eye-tracking data. Researchers should plan to collect large samples of participants for online experiments, which of course is strongly facilitated given that an online study can test multiple participants at the same time (subject to platform limitations) and does not require the presence or attention of an experimenter.

Based on our experience running lab-based sessions, we found that certain aspects of the set-up tended to support cleaner, more reliable tracking: the light and position are key to the data collection, including diffused lighting facing the participant, positioned behind the computer or webcam rather than overhead or behind them, which avoided glare and backlighting; a webcam positioned at the top of the screen, at a distance of approximately 40–50 cm from the participant; the computer placed on a stable surface; and an upright seated posture with minimal head movement or talking during the session. For online studies, simple instructions to participants, such as cleaning the webcam lens before starting, a room without distractions, and ensuring there is adequate lighting behind the computer to illuminate their face, could make a meaningful difference. While these observations come from controlled laboratory conditions rather than a systematic test of their individual contribution to online data quality, they may offer a useful starting point for researchers designing future online webcam studies.

Our findings demonstrate that webcam-based eye tracking, as implemented in the Labvanced platform, can successfully capture both spatial gaze patterns and word-level cognitive effects during sentence reading, including the classical word frequency effect. These results are consistent with, and extend, previous work showing that webcam eye tracking can detect robust cognitive effects when regions of interest are sufficiently large or well separated (Slim & Hartsuiker, 2023; Prystauka et al., 2023; Slim et al., 2024; Kandel & Snedeker, 2025). Similarly, Kaduk et al. (2023) reported high positional correlations between Labvanced and EyeLink data in attention tasks, but their study did not address reading; our findings extend this work by demonstrating that Labvanced can also detect word-level reading effects. Furthermore, our results provide empirical support for Angele et al.’s (2025) prediction that lexical frequency effects should remain detectable even at sampling rates as low as 30–60 Hz. Although webcam eye tracking is limited in its ability to provide the high spatial precision required for certain cognitive tasks involving fine-grained gaze discrimination (James et al., 2025), our findings indicate that, when noise is mitigated through increased observations and appropriate design adaptations, webcam-based tracking can nevertheless be adequate for detecting word-level effects during sentence reading.

Still, fixation durations detected by webcams may differ substantially from those detected by high-precision eye trackers. In particular, the samples from the webcam signal were grouped into fewer, but longer fixations (see Appendix A). This mostly occurs because the lower spatial precision of webcams makes small saccades difficult to distinguish from positional noise, resulting in fewer detected saccades and the merging of adjacent fixations. Even at a sampling rate of 60 Hz (a new sample every 16.7 ms), many reading saccades lasting ~20–70 ms are represented by only 1–4 samples. Therefore, if the positional displacement of short saccades is indistinguishable from spatial noise, their detection can be challenging. This has a couple of implications. First, the average “benchmark” fixation durations observed in the lab (225–250 ms; Rayner, 2009) may not apply to webcam eye tracking, and researchers may need to come up with new reference values and data cleaning cut-offs. Second, because adjacent fixation segments are more likely to be merged, webcam eye tracking may be less suitable for testing effects or computational models that depend on the precise timing of oculomotor events. Rather, it would be better suited for detecting relative differences between conditions or participant populations, especially for larger effects which are also not very time-sensitive. This fixation-merging pattern may partly contribute to the higher skipping probability observed in the webcam data relative to EyeLink across experiments, and is worth considering when interpreting skipping-based measures obtained with webcam eye tracking.

In the future, additional experimental platforms and experimental designs should be evaluated. For example, would it be possible to record participants reading larger discourse materials consisting of multiple paragraphs of text in an online experiment and still obtain useful data? The challenge there would lie in striking a balance between using large font sizes and fitting enough text on the screen to study discourse processes. One solution could be to present larger texts one sentence at a time, in a “self-paced reading” style of presentation. This approach would reduce the naturalness of reading (e.g., prevent long regressions), but would still preserve accuracy. Alternatively, font size could be reduced to fit more text, but sample sizes would need to be increased to counter the increase in error.

Given its lower precision, we are unsure whether our experimental approach would work with WebGazer (Papoutsaki et al., 2016), but it is possible that useful information could be obtained with very large text and a very large sample of participants. We recommend that researchers who wish to use a different platform for their studies do a pilot study first, demonstrating the feasibility of detecting a well-established effect such as the word frequency effect. If the size of the effect of interest is smaller than the size of the word frequency effect observed in the present study, online eye tracking may not be an adequate technique to use. Nevertheless, we encourage researchers to investigate statistical power in the context of online eye tracking during reading and to attempt to provide an estimate of the smallest effect sizes that can realistically be detected using this technique.

More broadly, we believe that webcam-based eye tracking is currently best positioned as a complementary method whose near-term theoretical contribution is likely to lie more in extending and replicating robust findings than in adjudicating between fine-grained theoretical accounts. Under the current technical constraints, webcam-based approaches may be especially useful for replicating robust benchmark effects, testing well-established predictions in less-studied languages or populations, and scaling up data collection when large samples or broader accessibility are important. By contrast, questions that depend on subtle temporal dynamics, very small effect sizes, or fine-grained spatial discrimination are likely to remain better suited to high-precision laboratory eye trackers. We therefore see webcam eye tracking as a complementary methodological tool that can broaden the empirical reach of reading research, while being used with caution (or not at all) when strong theoretical inferences depend on precise eye-movement measures. We want to outline more specific considerations to help guide this decision in practice.

First, task characteristics are important to decide whether to use webcam-based eye tracking. The need for large font sizes and short lines to support reliable tracking constrains the type of materials that can be used, making single sentences more straightforward than longer texts or paragraphs. Sampling rate is also related to this decision: the low sampling rate of webcams (30 Hz for most consumer devices) would not be ideal for research requiring fine-grained temporal resolution, such as the duration of very short fixations, or paradigms relying on gaze-contingent display changes (e.g., the boundary paradigm or moving window paradigm). Detecting a saccade in time to trigger an invisible display change would require a sampling rate above 30 Hz, since a saccade lasting 20–70 ms may already be complete before the next sample is even recorded. A related limitation is that fewer fixations tend to be detected, and those that are detected tend to be longer on average, with implications for measures such as skipping probability, which could be overestimated relative to EyeLink-based studies, and for any analyses that depend on accurately detecting short inter-word movements. Finally, cumulative dependent measures such as total viewing time and gaze duration, which aggregate fixation time across multiple fixations, are likely to be more robust in webcam data than single-fixation measures such as first fixation duration or single fixation duration, which rely on the accurate parsing of individual short fixations. Overall, webcam eye tracking seems best suited for studies that prioritise broad participant access and large sample sizes over fine-grained spatial and temporal precision.

We hope that our results will encourage other researchers to attempt eye-tracking experiments using webcams, while keeping in mind the sample size requirements. Importantly, webcam research does not necessarily have to be performed completely online: a researcher wishing to study eye tracking and requiring slightly higher precision than that available online might run a webcam experiment on a laptop or a computer with an external webcam, as we did in Experiments 1 and 2. By using an external webcam, the quality of the data may be further enhanced. Both online and in the laboratory, our results make us optimistic about the potential for employing webcam eye tracking to aid the study of reading in places and with populations where high-precision eye tracker access is difficult.

## Conclusion

In summary, our results clearly demonstrate that it is possible to obtain useful eye-tracking data about the processing of individual words during sentence reading in an online experiment, at least using the experimental design and the online platform (Labvanced) that we employed. We hope that webcam eye tracking will complement lab-based eye trackers as an additional method for obtaining useful data about language processing during reading, particularly in difficult-to-reach populations. At present, its near-term theoretical value may lie more in enabling robust effects to be tested and replicated across a wider range of languages, populations, and research settings than in resolving fine-grained mechanistic debates.

## Electronic supplementary material

Below is the link to the electronic supplementary material.

Supplementary file1 (DOCX 238 KB)

Supplementary file2 (DOCX 2311 KB)

## Figures and Tables

**Fig. 9:**
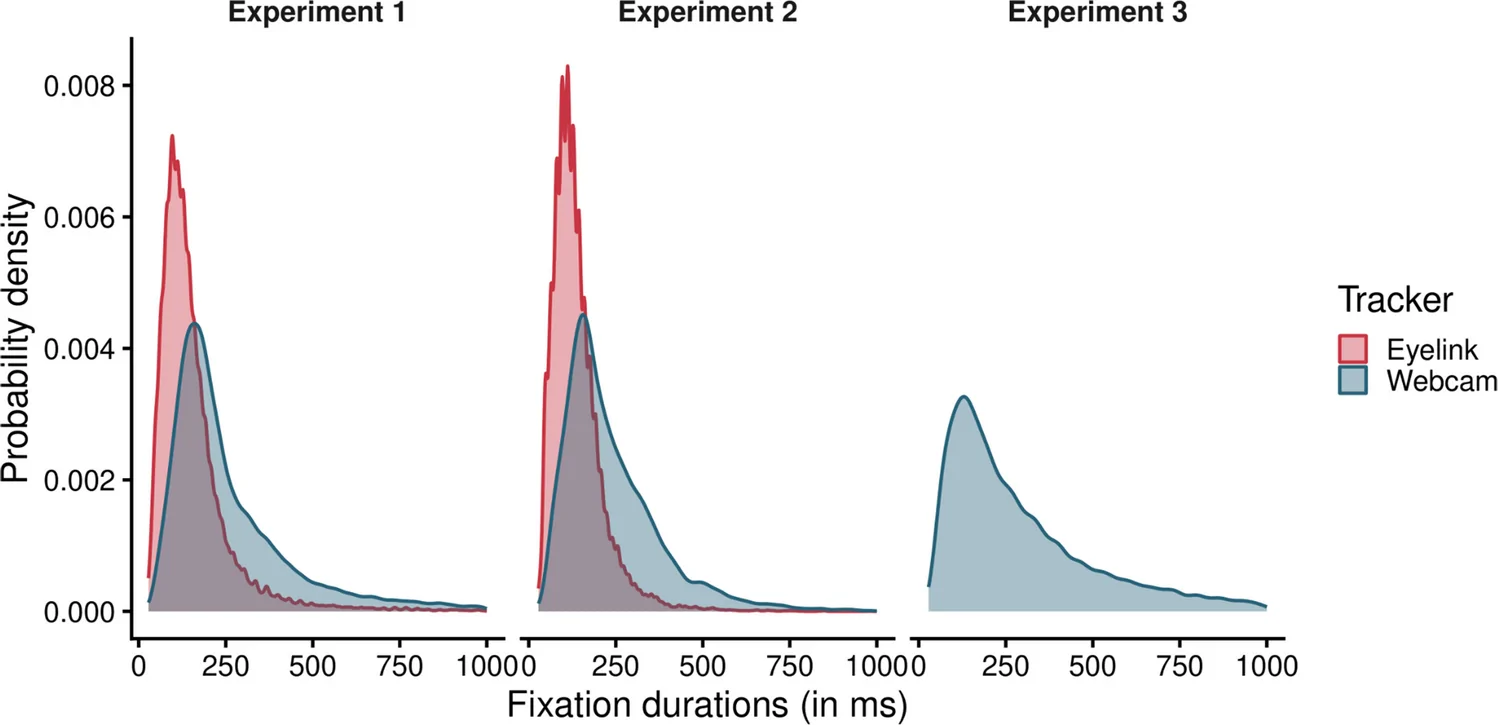
Distribution of fixation durations detected from the raw sample data using the Engbert and Kliegl (2003) algorithm. The data from Experiments 1 and 3 contain both the Frequency and Single-line corpus combined. Note that fixation durations > 1,000 ms were trimmed for visualisation purposes (1.02% of all data)

## Appendix

Figs. 9 and 10

## Footnotes

1 Eight other participants were tested, but their data were discarded for the following reasons: three due to poor calibration accuracy, four due to technical issues with the webcam recording, and one due to not being a fluent reader. One participant (#17) contributed data only to the Frequency corpus due to webcam recording issues in the second half of the study.

2 It should be noted that, as far as we can tell, Kaduk et al. (2023) did not use a chinrest for recording. We opted to do so to minimise head movements and increase recording accuracy.

3 There were more fixations < 80 ms detected for the EyeLink data than the webcam data. Because the webcam data are noisier, fewer small saccades are detected by the algorithm. As a result, the data tend to be grouped into fewer, but longer, fixations. The EyeLink data, on the other hand, have a more spatially consistent signal, which makes the detection of more of these smaller saccades possible. While this can be addressed by increasing the detection threshold (λ) for the EyeLink data, we opted not to do so in the spirit of treating both signals in the same way.

4 The versions of R and all packages used are as follows: R (Version 4.5.2; R Core Team, 2025) and the R-packages *conflicted* (Version 1.2.0; Wickham, 2023), *dplyr* (Version 1.1.4; Wickham et al., 2023), *emmeans* (Version 2.0.0; Lenth & Piaskowski, 2025), *forcats* (Version 1.0.1; Wickham, 2025a, 2025b), *ggplot2* (Version 4.0.0; Wickham, 2016), *gt* (Version 1.1.0; Iannone et al., 2025), *here* (Version 1.0.2; Müller, 2025), *knitr* (Version 1.50; Xie, 2015), *lubridate* (Version 1.9.4; Grolemund & Wickham, 2011), *modelsummary* (Version 2.5.0; Arel-Bundock, 2022), *papaja* (Version 0.1.4; Aust & Barth, 2022), *purrr* (Version 1.2.0; Wickham & Henry, 2025), *qs2* (Version 0.1.5; Ching, 2025), *readr* (Version 2.1.5; Wickham et al., 2024a, 2024b), *sjPlot* (Version 2.9.0; Lüdecke, 2025), *stringr* (Version 1.6.0; Wickham, 2025b), *tibble* (Version 3.3.0; Müller & Wickham, 2025a, 2025b), *tidybayes* (Version 3.0.7; Kay, 2024), *tidyr* (Version 1.3.1; Wickham, Vaughan, et al., 2024), *tidyverse* (Version 2.0.0; Wickham et al., 2019), and *tinylabels* (Version 0.2.5; Barth, 2025).

5 Experiment 3 still employed the same set-up of presenting the lexical frequency items over 3–4 lines of text. Because Experiments 2 and 3 were running largely in parallel, we could not use the previous findings to inform our design decisions. However, the advantage of this is that Experiment 3 provides a direct replication of Experiment 1 in the real world.

6 The webcam interface does not record the sampling rate of the webcam that participants had. However, based on the effective sampling rate (calculated on the raw data), it can be deduced that almost all participants had a 30 Hz web camera. The effective sampling rate is a bit lower than the expected rate of 30 Hz. While this is partly due to the software not achieving the full sampling rate (which was also evident in Experiments 1 and 2), the greater variance in effective sampling rate between participants may be due to differences in internet speed, which can affect the speed at which samples are sent to the server. Post hoc analysis suggested that effective sampling rate does not modulate the target word lexical frequency effect.

## Declarations

### Open practices statement

Data, code, and materials from the three studies are publicly available at: https://osf.io/axq7u/. None of the experiments were pre-registered.
