# Supplementary material for: Webcams can be used to study eye movements during reading
Source: Behav Res Methods. 2026 Oct 21;58(12):331. doi: 10.3758/s13428-026-03199-3 (PMC13687295; doi:10.3758/s13428-026-03199-3)
Supplement: Supplementary file 1 [file 13428_2026_3199_MOESM1_ESM.docx]

**Supplementary Materials**

**Main Results from Experiments 1-2 (Downsampled to 30 Hz)**

The data from Experiments 1 and 2 were natively recorded at 60 Hz sampling rate. However, because 60 Hz webcams are not (yet) widely available, this raises the question of how representative these results are for data collection in the real world. While Experiment 3 provides some evidence that useful data can still be obtained even with 30 Hz cameras, there is still some value in testing whether similar results would be obtained in Experiments 1-2, *had* the data been collected at 30 Hz. To simulate this, we downsampled the 60 Hz data to 30 Hz (by taking every second sample) and repeated the main target word lexical frequency analyses.

**Experiment 1**. Descriptive statistics from the downsampled data in Experiment 1 are shown in Table S1 and the (G)LMM results are shown in Tables S2. The downsampled Eyelink and webcam data exhibited correlations of similar magnitude to the 60Hz data reported in the main paper (see Figure S1).

*Figure S1*. Pearson’s *r* correlations for the 30Hz downsampled data between the Eyelink and webcam gaze sample position in Experiments 1 and 2, calculated separately for the x- and y-dimension. Each dot represents the average correlation for a participant in the study. The black dot represents the average across all participants.

Table S1

*Mean first fixation duration (FFD), single fixation duration (SFD), gaze duration (GD), total viewing time (TVT), and first-pass skipping probability on target words in Experiment 1, using downsampled data at 30 Hz, grouped by eye-tracker and target word frequency condition.*

|  |  | FFD | | SFD | | GD | | TVT | | Skipping probability | |
| --- | --- | --- | --- | --- | --- | --- | --- | --- | --- | --- | --- |
| Tracker | Frequency | Mean | SD | Mean | SD | Mean | SD | Mean | SD | Mean | SD |
| Webcam | low | 300 | 201 | 303 | 203 | 357 | 255 | 501 | 362 | 0.57 | 0.49 |
| Webcam | high | 274 | 187 | 271 | 181 | 335 | 252 | 480 | 389 | 0.59 | 0.49 |
| Eyelink | low | 293 | 194 | 302 | 198 | 325 | 212 | 398 | 267 | 0.29 | 0.45 |
| Eyelink | high | 251 | 168 | 255 | 168 | 270 | 182 | 337 | 242 | 0.33 | 0.47 |
| *Note*: All means and standard deviations are reported in milliseconds (ms). | | | | | | | | | |  |  |

There was a credible target word frequency effects in all measures, with fixation durations being longer and skipping probability being lower on low compared to high frequency target words (FFD: $b=0.10$, 95% CrI $\left[ 0.05, 0.15 \right]$, $P\left( b>0 \right)=1.00$, $ER_{b>0}>1000$, $BF_{10}=484.35$; SFD: $b=0.11$, 95% CrI $\left[ 0.06, 0.17 \right]$, $P\left( b>0 \right)=1.00$, $ER_{b>0}>1000, BF_{10}=28.90$; GD: $b=0.10$, 95% CrI $\left[ 0.05, 0.16 \right]$, $P\left( b>0 \right)=1.00$, $ER_{b>0}>1000$, $BF_{10}=17.81$; TVT: $b=0.11$, 95% CrI $\left[ 0.05, 0.17 \right]$, $P\left( b>0 \right)=1.00$, $ER_{b>0}>1000$, $BF_{10}=7.51;$ Skipping: $b=-0.18$, 95% CrI $\left[ -0.34, -0.01 \right]$, $P\left( b<0 \right)=0.98$, $ER_{b<0}=59.38$, $BF_{10}=0.78$).

The effect of Tracker was credible in FFD ($b=0.09$, 95% CrI $\left[ 0.007, 0.17 \right]$, $P\left( b>0 \right)=0.98$, $ER_{b>0}=53.98$, $BF_{10}=0.44$), GD ($b=0.17$, 95% CrI $\left[ 0.09, 0.25 \right]$, $P\left( b>0 \right)=1.00$, $ER_{b>0}>1000$, $BF_{10}=103.62$), TVT ($b=0.29$, 95% CrI $\left[ 0.19, 0.40 \right]$, $P\left( b>0 \right)=1.00$, $ER_{b>0}>1000$, $BF_{10}>1000$), and skipping probability ($b=1.63$, 95% CrI $\left[ 1.12, 2.17 \right]$, $P\left( b>0 \right)=1.00$, $ER_{b>0}>1000$, $BF_{10}>1000$), though the effect in SFD included 0 in the credible interval ($b=0.07$, 95% CrI $\left[ -0.02, 0.15 \right]$, $P\left( b>0 \right)=0.93$, $ER_{b>0}=13.81$, $BF_{10}=0.14$). Similar to the results in the main paper, this was due to fixation durations being longer and skipping probability being higher in the webcam compared to the Eyelink data. Finally, the Frequency x Tracker interaction was credible in GD ($b=-0.14$, 95% CrI $\left[ -0.25, -0.04 \right]$, $P\left( b<0 \right)=1.00$, $ER_{b<0}=300.89$, $BF_{10}=1.89$) and TVT ($b=-0.14$, 95% CrI $\left[ -0.25, -0.03 \right]$, $P\left( b<0 \right)=0.99$, $ER_{b<0}=152.85$, $BF_{10}=1.15$), but not in the remaining measures (FFD: $b=-0.08$, 95% CrI $\left[ -0.18, 0.02 \right]$, $P\left( b<0 \right)=0.95$, $ER_{b<0}=17.63$, $BF_{10}=0.18$; SFD: $b=-0.10$, 95% CrI $\left[ -0.20, 0.005 \right]$, $P\left( b<0 \right)=0.97$, $ER_{b<0}=30.07$, $BF_{10}=0.29$; Skipping: $b=0.14$, 95% CrI $\left[ -0.21, 0.47 \right]$, $P\left( b>0 \right)=0.79$, $ER_{b>0}=3.72$, $BF_{10}=0.24$). The two significant interactions were due to the Frequency effect being stronger in the Eyelink compared to the webcam data.

The marginal means showed a similar pattern to the results in the main paper. While the Frequency effect was credible in the Eyelink data for all measures, it was not credible in the the webcam data for any of them (FFD: Webcam: *M* = 0.06 95% *CrI* = [-0.02, 0.14]; Eyelink: *M* = 0.14 95% *CrI* = [0.08, 0.20]; SFD: Webcam: *M* = 0.06 95% *CrI* = [-0.02, 0.15]; Eyelink: *M* = 0.16 95% *CrI* = [0.10, 0.23]; GD: Webcam: *M* = 0.03 95% *CrI* = [-0.06, 0.12]; Eyelink: *M* = 0.17 95% *CrI* = [0.11, 0.24]; TVT: Webcam: *M* = 0.04 95% *CrI* = [-0.05, 0.14]; Eyelink: *M* = 0.18 95% *CrI* = [0.11, 0.25]; Skipping: Webcam: *M* = -0.11 95% *CrI* = [-0.36, 0.14]; Eyelink: *M* = - 0.25 95% *CrI* = [-0.47, -0.03]). Nonetheless, both the webcam and Eyelink data showed effects in the same direction and similar magnitude (except GD and TVT where the effect was weaker in the webcam data). In summary, despite some minor differences and the presence of a few interactions, the main results from Experiment 1 were replicated when using 30 Hz downsampling and the conclusions remain unchanged.

Table S2

*Bayesian linear mixed effects models showing the target word lexical frequency effect in Experiment 1 (using 30 Hz downsampled data). The Results show coefficient estimates and 95% credible intervals (in square brackets).*

|  | FFD | SFD | GD | TVT | Skipping probability |
| --- | --- | --- | --- | --- | --- |
| Intercept | **5.473** | **5.485** | **5.593** | **5.827** | -0.208 |
|  | [**5.369, 5.575**] | [**5.382, 5.592**] | [**5.488, 5.696**] | [**5.727, 5.928**] | [-0.589, 0.183] |
| Frequency | **0.101** | **0.113** | **0.102** | **0.113** | **-0.177** |
|  | [**0.051, 0.150**] | [**0.059, 0.166**] | [**0.048, 0.156**] | [**0.049, 0.175**] | [**-0.342, -0.014**] |
| Tracker | **0.089** | 0.065 | **0.170** | **0.293** | **1.634** |
|  | [**0.007, 0.170**] | [-0.021, 0.150] | [**0.086, 0.254**] | [**0.189, 0.399**] | [**1.116, 2.170**] |
| Frequency × Tracker | 0.080 | -0.098 | **-0.143** | **-0.138** | 0.140 |
|  | [-0.175, 0.017] | [-0.203, 0.005] | [**-0.247, -0.041**] | [**-0.249, -0.030**] | [-0.210, 0.471] |

*Note*: Effects for which the 95% CrI excludes 0 are highlighted in bold. FFD: *first fixation duration; SFD: single fixation duration; GD: gaze duration; TVT: total viewing time.*

**Experiment 2**.

Descriptive statistics from the downsampled data in Experiment 2 are provided in Table S3 and the (G)LMM results are provided in Table S4.

Table S3

*Mean first fixation duration (FFD), single fixation duration (SFD), gaze duration (GD), total viewing time (TVT), and first-pass skipping probability on target words in Experiment 2, using downsampled data at 30 Hz, grouped by eye-tracker and target word frequency condition.*

|  |  | FFD | | SFD | | GD | | TVT | | Skipping probability | |
| --- | --- | --- | --- | --- | --- | --- | --- | --- | --- | --- | --- |
| Tracker | Frequency | Mean | SD | Mean | SD | Mean | SD | Mean | SD | Mean | SD |
| Webcam | low | 253 | 163 | 255 | 163 | 265 | 176 | 296 | 201 | 0.42 | 0.49 |
| Webcam | high | 236 | 144 | 236 | 143 | 243 | 150 | 272 | 188 | 0.43 | 0.50 |
| Eyelink | low | 239 | 160 | 242 | 161 | 249 | 166 | 266 | 176 | 0.36 | 0.48 |
| Eyelink | high | 224 | 135 | 223 | 135 | 232 | 148 | 251 | 168 | 0.37 | 0.48 |
| *Note*: All means and standard deviations are reported in milliseconds (ms). | | | | | | | | | |  |  |

Similar to the 60 Hz data, there was a credible target word frequency effects, with fixation durations being longer on low compared to high frequency target words (FFD: $b=0.07$, 95% CrI $\left[ 0.02, 0.13 \right]$, $P\left( b>0 \right)=1.00$, $ER_{b>0}=230.88$, $BF_{10}=0.90$; SFD: $b=0.08$, 95% CrI $\left[ 0.02, 0.14 \right]$, $P\left( b>0 \right)=1.00$, $ER_{b>0}=249, BF_{10}=1.14$; GD: $b=0.08$, 95% CrI $\left[ 0.03, 0.14 \right]$, $P\left( b>0 \right)=1.00$, $ER_{b>0}=409.26$, $BF_{10}=1.48$; TVT: $b=0.09$, 95% CrI $\left[ 0.03, 0.15 \right]$, $P\left( b>0 \right)=1.00$, $ER_{b>0}=499.0$, $BF_{10}=2.18$). However, there was generally no effect in skipping probability ($b=0.005$, 95% CrI $\left[ -0.37, 0.36 \right]$, $P\left( b>0 \right)=0.51$, $ER_{b>0}=1.05$, $BF_{10}=0.18$), suggesting that this measure is not captured well in the 30Hz data.

The effect of Tracker was also credible for all fixation duration measures (FFD: $b=0.07$, 95% CrI $\left[ 0.02, 0.12 \right]$, $P\left( b>0 \right)=0.99$, $ER_{b>0}=178.78$, $BF_{10}=0.95$; SFD: $b=0.08$, 95% CrI $\left[ 0.03, 0.13 \right]$, $P\left( b>0 \right)=1.00$, $ER_{b>0}=431.43$, $BF_{10}=1.89$; GD: $b=0.08$, 95% CrI $\left[ 0.03, 0.13 \right]$, $P\left( b>0 \right)=1.00$, $ER_{b>0}=760.9$, $BF_{10}=3.19$; TVT: $b=0.09$, 95% CrI $\left[ 0.04, 0.14 \right]$, $P\left( b>0 \right)=1.00$, $ER_{b>0}>9.06, BF_{10}=9.06$), but not skipping probability, where there was again no clear evidence of a difference ($b=0.33$, 95% CrI $\left[ -0.09, 0.74 \right]$, $P\left( b>0 \right)=0.94$, $ER_{b>0}=16.13$, $BF_{10}=0.74$). The interaction between Tracker and Frequency was also not credible for any of the measures (FFD: $b=0.02$, 95% CrI $\left[ -0.07, 0.10 \right]$, $P\left( b>0 \right)=0.65$, $ER_{b>0}=1.82$, $BF_{10}=0.04$; SFD: $b=0.01$, 95% CrI $\left[ -0.07, 0.09 \right]$, $P\left( b>0 \right)=0.59$, $ER_{b>0}=1.44$, $BF_{10}=0.04$; GD: $b=0.02$, 95% CrI $\left[ -0.06, 0.10 \right]$, $P\left( b>0 \right)=0.67$, $ER_{b>0}=2.07$, $BF_{10}=0.04$; TVT: $b=0.02$, 95% CrI $\left[ -0.05, 0.10 \right]$, $P\left( b>0 \right)=0.72$, $ER_{b>0}>2.56, BF_{10}=0.05$; Skipping: $b=-0.04$, 95% CrI $\left[ -0.34, 0.26 \right]$, $P\left( b<0 \right)=0.60$, $ER_{b<0}=1.49$, $BF_{10}=0.16$).

The marginal means in Experiment 2 also showed a similar pattern to the results in the main paper (FFD: Webcam: *M* = 0.08 95% *CrI* = [0.01, 0.15]; Eyelink: *M* = 0.07 95% *CrI* = [-0.001, 0.13]; SFD: Webcam: *M* = 0.08 95% *CrI* = [0.01, 0.16]; Eyelink: *M* = 0.08 95% *CrI* = [0.01, 0.14]; GD: Webcam: *M* = 0.09 95% *CrI* = [0.02, 0.16]; Eyelink: *M* = 0.07 95% *CrI* = [0.01, 0.14]; TVT: Webcam: *M* = 0.10 95% *CrI* = [0.03, 0.17]; Eyelink: *M* = 0.08 95% *CrI* = [0.01, 0.15]; Skipping: Webcam: *M* = -0.02 95% *CrI* = [-0.40, 0.35]; Eyelink: *M* = 0.02 95% *CrI* = [-0.40, 0.42]). The frequency effect was credible in all fixation duration measures for both the Eyelink and the Webcam (with the only exception that the Eyelink effect in FFD narrowly included 0). However, the effect in skipping was not credible again for both the Eyelink and Webcam data. In summary, the results show that downsampling the Experiment 2 data to 30 Hz does not fundamentally change the results, suggesting that the target word frequency effects are still present in fixation durations.

Table S4

*Bayesian linear mixed effects models showing the target word lexical frequency effect in Experiment 2 (using 30 Hz downsampled data). The Results show coefficient estimates and 95% credible intervals (in square brackets).*

|  | FFD | SFD | GD | TVT | Skipping probability |
| --- | --- | --- | --- | --- | --- |
| Intercept | **5.309** | **5.321** | **5.340** | **5.401** | **-0.508** |
|  | [**5.229, 5.390**] | [**5.237, 5.406**] | [**5.251, 5.427**] | [**5.310, 5.490**] | [**-0.821, -0.188**] |
| Frequency | **0.073** | **0.080** | **0.083** | **0.091** | 0.005 |
|  | [**0.019, 0.128**] | [**0.023, 0.139**] | [**0.025, 0.141**] | [**0.031, 0.150**] | [-0.367, 0.358] |
| Tracker | **0.073** | **0.078** | **0.080** | **0.095** | 0.329 |
|  | [**0.021, 0.124**] | [**0.027, 0.129**] | [**0.030, 0.129**] | [**0.044, 0.144**] | [-0.087, 0.744] |
| Frequency × Tracker | 0.015 | 0.009 | 0.018 | 0.023 | -0.037 |
|  | [-0.068, 0.097] | [-0.070, 0.089] | [-0.061, 0.097] | [-0.055, 0.101] | [-0.341, 0.262] |

*Note*: Effects for which the 95% CrI excludes 0 are highlighted in bold. FFD: *first fixation duration; SFD: single fixation duration; GD: gaze duration; TVT: total viewing time.*
