# Supplementary material for: Webcams can be used to study eye movements during reading
Source: Behav Res Methods. 2026 Oct 21;58(12):331. doi: 10.3758/s13428-026-03199-3 (PMC13687295; doi:10.3758/s13428-026-03199-3)
Supplement: Supplementary file 2 [file 13428_2026_3199_MOESM2_ESM.docx]

**Appendix A**

*Figure A1*. Distribution of fixation durations detected from the raw sample data using the Engbert and Kliegl (2003) algorithm. The data from Experiments 1 and 3 contains both the Frequency and Single-line corpus combined. Note that fixation durations > 1000 ms were trimmed for visualisation purposes (1.02% of all data).

**Appendix B**

*Figure B1*. Power spectrum plots for the webcam data based on the results from Experiment 3. Power levels were computed for different subject and item numbers (ranging between 10 and 150, in intervals of 10). The 80% power level is indicated by a solid black contour lines. Simulations were conducted with the *simr* package [(Green & MacLeod, 2016)](https://www.zotero.org/google-docs/?Grmhsq). Power was calculated with 100 simulations per cell and included 10% random data loss. The models included random intercepts for subjects and items, and random slopes for target word frequency.
